# Identification and Characterization of Genes Encoding the Hydroxypyruvate Reductases in *Chlamydomonas* Reveal Their Distinct Roles in Photorespiration

**DOI:** 10.3389/fpls.2021.690296

**Published:** 2021-06-24

**Authors:** Menglin Shi, Lei Zhao, Yong Wang

**Affiliations:** ^1^College of Life Sciences, Nankai University, Tianjin, China; ^2^Key Laboratory of Systems Microbial Biotechnology, Tianjin Institute of Industrial Biotechnology, Chinese Academy of Sciences, Tianjin, China; ^3^National Center of Technology Innovation for Synthetic Biology, Tianjin, China

**Keywords:** *Chlamydomonas*, photorespiration, photosynthesis, hydroxypyruvate reductase, glycolate

## Abstract

Photorespiration plays an important role in maintaining normal physiological metabolism in higher plants and other oxygenic organisms, such as algae. The unicellular eukaryotic organism *Chlamydomonas* is reported to have a photorespiration system different from that in higher plants, and only two out of nine genes encoding photorespiratory enzymes have been experimentally characterized. Hydroxypyruvate reductase (HPR), which is responsible for the conversion of hydroxypyruvate into glycerate, is poorly understood and not yet explored in *Chlamydomonas*. To identify the candidate genes encoding hydroxypyruvate reductases in *Chlamydomonas* (CrHPR) and uncover their elusive functions, we performed sequence comparison, enzyme activity measurement, subcellular localization, and analysis of knockout/knockdown strains. Together, we identify five proteins to be good candidates for CrHPRs, all of which are detected with the activity of hydroxypyruvate reductase. CrHPR1, a nicotinamide adenine dinucleotide (NADH)-dependent enzyme in mitochondria, may function as the major component of photorespiration. Its deletion causes severe photorespiratory defects. CrHPR2 takes part in the cytosolic bypass of photorespiration as the compensatory pathway of CrHPR1 for the reduction of hydroxypyruvate. CrHPR4, with NADH as the cofactor, may participate in photorespiration by acting as the chloroplastidial glyoxylate reductase in glycolate-quinone oxidoreductase system. Therefore, the results reveal that CrHPRs are far more complex than previously recognized and provide a greatly expanded knowledge base for studies to understand how CrHPRs perform their functions in photorespiration. These will facilitate both modification of photorespiration and genetic engineering for crop improvement by synthetic biology.

## Introduction

Photosynthesis and photorespiration are the two major pathways of plant primary metabolism mediated by bifunctional enzyme ribulose bisphosphate carboxylase/oxygenase (Rubisco; Griffiths, 2006). Since cellular O_2_ concentration is much higher than that of CO_2_, a correspondingly high amount of photosynthetically fixed carbon is released *via* the oxidation of Rubisco during photorespiration (Somerville, 2001). The loss of fixed carbon associated with photorespiration could be greatly elevated by rising temperature and drought, resulting in a severe reduction in crop yields (Bauwe et al., 2012). Hence, photorespiration is becoming an important target for crop improvement and obtaining more attention, as the world will face more serious challenges, such as extreme climate and severe food shortage (Ort et al., 2015).

Photorespiration, conserved in higher plants and most algae, comprises a series of nine consecutive enzymatic reactions distributed over chloroplasts, peroxisomes, mitochondria, and the cytosol (Bauwe et al., 2012). It starts from the catalysis mediated by Rubisco, in which ribulose 1,5-bisphosphate (RuBP) is oxidized into 2-phosphoglycolate (2-PG) and 3-phosphoglycerate (3-PGA, Tcherkez, 2013). 2-PG is dephosphorylated by phosphoglycolate phosphatase, leading to the generation of glycolate, which enters photorespiratory metabolism flux for further conversion (Douce and Heldt, 2000). In the penultimate step of the photorespiration cycle, hydroxypyruvate is converted to glycerate catalyzed by hydroxypyruvate reductase (HPR), and eventually to Calvin–Benson intermediate 3-PGA (Hagemann and Bauwe, 2016). Despite the good consensus on the photorespiratory cycle, serial catalytic enzymes and their detailed functions have not yet been globally characterized until now (Eisenhut et al., 2019). One question that remains to be resolved is concerning the presence and function of HPR in *Chlamydomonas* photorespiration, which is the topic of this study.

Hydroxypyruvate reductases are highly conserved evolutionarily; however, they are only experimentally characterized to a limited extent, especially in terms of an underlying gene at the molecular level. Since the 1970s, HPRs have been purified and studied by their enzymological property with then state-of-the-art methodology, and the studies mainly focused on HPRs from barley (Kleczkowski et al., 1990), spinach (Kleczkowski and Randall, 1988; Kleczkowski et al., 1991), cucumber (Titus et al., 1983; Greenler et al., 1989; Schwartz et al., 1992), pumpkin (Hayashi et al., 1996; Mano et al., 2000), *Arabidopsis* (Mano et al., 1997; Cousins et al., 2008), and *Chlamydomonas* (Stabenau, 1974; Husic and Tolbert, 1987). Until recently, functional details of the underlying genes are revealed in model organisms, such as *Arabidopsis*. Deletion of HPR1 causes no visible alternation of growth or photorespiration in atmospheric air, differentiating from the lethal phenotype displayed by mutants with impairment in other photorespiratory components (Murray et al., 1989; Timm et al., 2008; Cousins et al., 2011; Ye et al., 2014). The non-typical photorespiratory phenotypes of hpr1 could be explained by a study on *Arabidopsis*, in which the absence of peroxisome-targeting AtHPR1 is partially compensated by the cytosolic bypass of photorespiration mediated by AtHPR2 (Timm et al., 2008; Li et al., 2019). Nevertheless, the combined deletion of both HPR1 and HPR2 does not result in the lethal phenotype, although typical photorespiratory characteristics were detected (Timm et al., 2008; Ye et al., 2014), suggesting the presence of additional hydroxypyruvate-reducing enzyme. By the basic local alignment search tool (BLAST) search, HPR3 was identified to be the potential candidate for such enzyme in *Arabidopsis*, and it showed activity with hydroxypyruvate using NADPH as the co-substrate (Timm et al., 2011). Interestingly, HPR3 could also accept glyoxylate as a substrate, suggesting that it may represent an additional bypass both to known HPRs and glyoxylate reductases in chloroplasts (Timm et al., 2011).

Unlike the higher plants, *Chlamydomonas* employs a CO_2_ concentration mechanism (CCM) to increase CO_2_ concentration in the vicinity of Rubisco, and its photorespiration system is assumed to work differently (Wang et al., 2015). Shortly after transfer from high to low CO_2_, but before the induction of CCM, photorespiration metabolism was induced rapidly and briefly (Brueggeman et al., 2012). Moreover, photorespiration is assumed to pass through mitochondria rather than peroxisome in *Chlamydomonas*, which is not yet globally studied and confirmed (Nakamura et al., 2005). In terms of HPR, Stabenau (1974) initially detected hydroxypyruvate reductases in *Chlamydomonas* (CrHPR) activity in mitochondria, then Husic and Tolbert (1985) further investigated enzymatic characteristics with cell extracts. In the decades since then, no further research on CrHPRs has been reported, and both the underlying genes and their detailed functions remain waiting to be discovered.

To search for the elusive CrHPRs, we have now exploited the *Chlamydomonas* genome and identified five proteins as candidates for being novel CrHPRs. Detailed studies indicate that all the five CrHPRs are detected with the activity of HPR. Using both bioinformatics and cell biology approaches, we were able to define the subcellular location of CrHPRs. We further addressed the physiological roles of CrHPR1, CrHPR2, and CrHPR4 by analyzing respective knockout/knockdown strains, and revealed their distinct roles in photorespiration. These findings are important steps toward understanding how CrHPRs perform their functions and will facilitate genetic engineering for crop improvement by providing prime targets for modification.

## Materials and Methods

### Strains and Culture Conditions

All *Chlamydomonas* strains used in the study are listed in Supplementary Table S1. The WT strain used was CC-125 (Chlamydomonas Resource Center).^1^ The mutant *Crhpr1* was generated as previously described (Cheng et al., 2017) and obtained from the Wu Han Jingyu Microalgae Science Co., Ltd., China. AmiRNA mutants were generated as previously described (Hu et al., 2014).

*Chlamydomonas* cells were maintained in solid Tris-acetate-phosphate (TAP) plates and cultured in liquid Tris-minimal medium (Gorman and Levine, 1965) at 25°C under 80 μE m^−2^ s^−1^ continuous light. For high CO_2_ treatment, cells were grown in Tris-minimal medium with aeration of 3% CO_2_.

### qRT-PCR Analysis

Total mRNAs used for qRT-PCR experiments were isolated from *Chlamydomonas* using the Eastep Super total RNA extraction kit (Promega, Madison, WI, United States), and cDNA was synthesized with the PrimeScript TMRT Master Mix kit (TaKaRa, Beijing). qRT-PCR reactions were performed in triplicate using Mastercycler ep realplex (Eppendorf, Hamburg, Germany) with gene-specific primers and actin as the internal control (Winck et al., 2016). Gene-specific PCR primer pairs used for the actin and CrHPRs are listed in Supplementary Table S2. The PCR primers were designed using Primer-BLAST of NCBI,^2^ and the amplifying program was as follows: pre-incubation at 95°C for 30 s, 40 cycles of denaturation at 95°C for 5 s, annealing at 6°C for 5 s, and amplification at 72°C for 25 s.

The change in fluorescence of SYBR Green I dye (SYBR Premix Ex Taq, TaKaRa) in every cycle was monitored by the realplex system software, and the cycle threshold (C_t_) above background for each reaction was calculated. The Ct value of actin was subtracted from that of the gene of interest to obtain aΔCt value. The Ct value of an arbitrary calibrator was subtracted from the ΔCt value to obtain a ΔΔCt value. The fold changes in expression level relative to the calibrator were calculated as 2^−ΔΔCt^.

### Gene Cloning, Heterologous Expression of *Chlamydomonas* Hydroxypyruvate Reductase, and Purification of Recombinant Proteins

Chlamydomonas cDNA samples were prepared as described above. HPR cDNAs were amplified using PrimeSTAR HS DNA Polymerase (Takara, Ohtsu, Japan) with gene-specific primers as listed in Supplementary Table S2. The PCR product was cloned directly into pMD20-T vector (Takara, Beijing, China), which was then transformed into competent *Escherichia coli* DH5α cells. Positive clones were verified by sequencing (BGI Genomics, Beijing, China).

For heterologous expression, plasmids containing HPR2 were digested with *Nco*I and *Xho*I, and the resulting fragment was linked to pETMALc-H treated with the same restricted enzymes (Pryor and Leiting, 1997), yielding pETMALc-H-HPR2. The plasmids containing CrHPR1, CrHPR3, CrHPR4, and CrHPR5 were digested with *Nde*I and *Xho*I, respectively, and then ligated into pET-30a digested with the same enzymes, yielding pET-30a-CrHPR1, pET-30a-CrHPR3, pET-30a-CrHPR4, and pET-30a-CrHPR5. The constructed expression plasmids were transformed into expression host *E. coli* Rosetta (DE3; CoWin Biosciences, Beijing, China), and the transformants were cultivated in the autoinduction medium ZYM2052 (Studier, 2005).

The recombinant HPRs were purified with HIS-Select nickel affinity gel filler (CoWin Biosciences, Beijing, China). Briefly, the supernatant of the broken cells were collected and gently mixed with HIS-Select nickel affinity gel, and then washed by three cycles of binding buffer. The His-tagged HPRs were eluted with elution buffer.

### Generation of amiRNA, Overexpression, and CrHPR-CFP Cell Lines

For the generation of amiRNA cell lines, a pHK460 vector was obtained from Kaiyao Huang (Institute of Hydrobiology, Chinese Academy of Sciences; Nordhues et al., 2012). Target gene-specific oligonucleotide sequences were designed using the WMD3 software.^3^ The resulting oligonucleotides that target CrHPR2 and CrHPR4 genes are listed in Supplementary Table S2. Combined with the sequence of miRNA cre-MIR1157, these oligonucleotides were linked into the pHK460 vector at the unique *Xho*I and *Eco*RI site. The plasmids were then isolated and subjected to transformation into *Chlamydomonas* cells.

For generation of overexpression cell lines, CrHPR1 was cloned from *Chlamydomonas* cDNA samples with gene-specific primers (*Crhpr1*-O-F/R), as shown in Supplementary Table S3. The PCR product was connected to the pMD20-T vector (Takara, Beijing, China) to form pMD20-CrHPR1, which was then transformed into competent *E. coli* DH5α cells. Positive clones were verified by sequencing (BGI Genomics, Beijing, China). The plasmid pMD20-CrHPR1 was digested with *Xho*I and *Eco*RI, and the resulting fragment was linked to pHK460 treated with the same restricted enzymes. The mRNA from pHK460 is expressed from the hsp70/rbcs2 promoter and ended at 3'UTR of /rbcs2. The transcript protein is fused with a Zeocin (Solarbio, Beijing) resistance selection maker, which will be cut off by the FMDV 2A self-cleaving sequence (Nordhues et al., 2012).

The constructed plasmids were transformed into *Chlamydomonas cells* using the electroporation method as described (Rasala et al., 2012). After transformation, the cells were grown on TAP agar supplemented with 15 μg/ml Zeocin (Sigma-Aldrich). Colonies derived from the single cells were picked for DNA extraction. After digestion with RNase, colonies were confirmed by PCR with primers (*Crhpr1*-O-F/R) to amplify the stripe, whose length is the same as that of the cDNA of CrHPR1.

For generation of CrHPR-mCerulean fluorescent protein (CFP) cell lines, CrHPRs were cloned from *Chlamydomonas* cDNA samples with gene-specific primers (CrHPRs-NT-F/R for testing N-terminal target signal and fusing the CFP to C-terminal of CrHPRs; CrHPRs-CT-F/R for testing C-terminal target signal and fusing the CFP to N-terminal of CrHPRs) as shown in Supplementary Table S2. The PCR products were connected into the pMD20-T vector (Takara, Beijing, China) to form pMD20-CrHPRs-NT or pMD20-CrHPRs-CT, and transformed into competent *E. coli* DH5α cells. Positive clones were verified by sequencing (BGI Genomics, Beijing, China). The plasmid pMD20-CrHPRs-NT was digested with *Xho*I and *Bam*HI, and the plasmid pMD20-CrHPRs-CT was digested with *Bam*HI and *Eco*RI. Then, the resulting fragment was linked to pHK460 treated with the same restricted enzymes.

The plasmids were then isolated and subjected to transformation into *Chlamydomonas* cells. Transformants were selected from TAP plates supplemented with 15 μg/ml Zeocin (Sigma-Aldrich).

### Fluorescence Microscopy

Representative cells were collected from TAP plates supplemented with Zeocin. Images were captured on Image-Pro Express 6.0 (Media Cybernetics, Rockville, MD, United States) using Olympus BX51 (Center Valley, PA, United States) with a Retiga-2000R camera (QImaging, Tucson, AZ, United States). The filters used in this research are CFP (excitation 436/10 nm, emission 470/30 nm) and Chloro (excitation 500/23 nm, emission 535/30 nm). The fluorescence images were false-colored using Adobe Photoshop CS3.

### Enzymatic Activity Assays

Cells were collected and broken in 1 ml extraction buffer (10 mM Tris-HCl, 1 mM EDTA, 2 mM MgCl_2_, 1 mM β-mercaptoethanol, pH 7.5), and the supernatant was collected for determination of enzymatic activity after centrifugation at 4°C. With 0.2 mM NADPH, 0.5 mM hydroxypyruvate or 1 mM glyoxylate, and 50 μl purified enzyme in 200 mM sodium phosphate buffer (pH 6.5). All reactions were started by the addition of 25–40 μg of a purified enzyme, and then the concentration was adjusted to make sure that the catalytic rate is proportional to reaction time during measurement time (5 min). In all the assays, one unit of enzyme activity was defined as 1 μmol of NADH converted per min. Hydroxypyruvate and glyoxylate reductase activity was determined according to published procedures (Husic and Tolbert, 1987) by measuring the absorbance of NAD(P)H at 340 nm using Microplate Photometer (Thermo-FC) at 25°C. The protein concentration was measured by Brandford method.

### Chlorophyll Fluorescence Measurement

Cells were immobilized, aiming to acquire accurate data, as described by Luz with modification (De-Bashan et al., 2015). Briefly, cells were cultured in a Tris-minimal medium and collected when the late logarithmic phase was reached. Then, the cells were washed with 0.85% (w/v) NaCl and concentrated 12 times, and 2 ml of the concentrated cells were mixed with 8 ml of 2% sodium alginate solution. The 4 cm^2^ squares of monofilament nylon with 24 threads per inch were sterilized and immersed into the above-mentioned mixture. The monofilament nylon square was quickly transferred into the 2% CaCl_2_ solution to solidify sodium alginate. The monofilament nylon squares were rinsed with 0.85% NaCl to remove excess solution. The above steps were repeated once again to get two layers of cell immobilized sodium alginate onto the squares.

The immobilized cells were clipped with a DLC-8 dark leaf clip and measured by MINI-PAM-II (Walz, Effeltrich, Germany) with the saturation pulse method by following the instruction of the manufacturer (Klughammer and Schreiber, 1994). Briefly, with the saturation pulse technique, the maximum quantum efficiency of PSII (Fv/Fm) was detected after 30-min dark acclimation. The light curves were generated by increasing actinic irradiance sequence ranging from 0 to 500 μmol photons m^−2^ s^−1^.

### Measurement of Photosynthetic and Respiration Rates

Cells at the late logarithmic phase (3–5 × 10^6^ cells/ml) were collected and concentrated five times. The oxygen exchange was measured with a Chlorolab-2 oxygen electrode (Hansatech, Norfolk, United Kingdom) at 30°C under both dark and light conditions by following the instructions of the manufacturer. Light intensity was determined using a quantum photometer (Hansatech, UK).

The electron flow of Rubisco was calculated by combining the data of oxygen exchange and chlorophyll fluorescence (Valentini et al., 1995). Formula: Jc = 1/3[ETRII + 8(A + R_D_)], Jo = 2/3[ETRII − 4(A + R_D_)], Jc: the electron flux of Rubisco carboxylation; Jo: the electron flux of Rubisco oxygenation; A: net photosynthetic rate; and R_D_: day respiration.

### Quantitative Measurement of Reactive Oxygen Species

The reactive oxygen species (ROS) of cells was measured by the ROS Assay Kit (Beyotime Institute of Biotechnology, Shanghai, China) following the instructions of the manufacturer. Briefly, cells at the late logarithmic stage were collected and stained with 100 μM 2',7'-dichlorofluorescein diacetate (H_2_DCF-DA) for 45 min at room temperature as described previously (Affenzeller et al., 2009). Then, the cells were washed three times with a Tris-minimal medium to remove the unbound probes and resuspend in a 1 ml Tris-minimal medium. The cell mixture was measured by fluorescence spectrophotometer (Enspire, PerkinElmer, Singapore) by 485 nm excitation and 530 nm emission.

### Glycolate Determination

Cells were collected at the late logarithmic phase and added to a new Tris-minimal medium to make the final concentration reach 2 × 10^6^ cells/ml. After 48 h, glycolate was detected from the supernatant according to the method of Kenji (Takahashi, 1972). Samples containing 0.1–10 μg glycolate were used to draw the standard curve, and 50 μl samples were added to the test tube with 1 ml 0.01% 2,7-dihydroxynaphthalene in concentrated sulfuric acid. After 20 min of 100°C water bath, the mixture was measured with Microplate Photometer (Thermo-FC) at 540 nm.

### Bioinformatic Analysis

To identify the *Chlamydomonas* HPR proteins, CrHPR1 protein sequence was searched against the *Chlamydomonas reinhardtii* predicted protein database in Phytozome 12 using the BLASTP function, searched against the HMMER *C. reinhardtii* reference proteome using the HMMER website service^4^ with the default parameters, or searched against AlgaePath.^5^ Protein alignments were performed using ClustalW (Larkin et al., 2007) and viewed using the GeneDoc software (Nicholas et al., 1997). The maximum likelihood phylogenetic tree was produced using the MEGA 7 program (Kumar et al., 2015).

## Results

### Identification and Bioinformatic Analysis of CrHPRs

Hydroxypyruvate reductase proteins are highly conserved over a wide range of species (Kutner et al., 2018). The putative *Chlamydomonas* HPR homolog is the protein encoded by *Cre06.g295450.t1.2* (hereafter designated as CrHPR1). The sequence alignment of HPR proteins from select species indicated the conservation of CrHPR1 as HPR, manifesting as the presence of highly conserved NAD(P)-binding motif, NAD recognition sites, substrate-orienting, and catalytic pair domains (Supplementary Figure S1).

To identify all candidate CrHPRs, the protein sequence of CrHPR1 was used to search against the *Chlamydomonas* proteome *via* the BLASTP function built on widely used online services, such as Phytozome 12, HMMER, and AlgaePath, with default parameters. The same list of eight proteins was retrieved with each of the three independent searches, and CrHPR1 itself was identified with the lowest E-value. Three remarkably similar proteins encoded by *Cre07.g344400*, *Cre07.g344550*, and *Cre07.g344600*, respectively, and annotated as phosphoglycerate dehydrogenase, were identified possibly because of the presence of NAD-binding and catalytic domains. Since they were reported to function in thylakoid membrane remodeling in response to adverse environmental conditions (Du et al., 2018), they are unlikely candidates for CrHPRs related to photorespiration and were not characterized further.

To confirm whether the proteins encoded by *Cre01.g019100.t1.2*, *Cre02.g087300.t1.3*, *Cre07.g324550.t1.3*, and *Cre16.g689700.t1.3* were authentic CrHPRs, sequence alignment was performed with CrHPR1 as the reference. As shown in Supplementary Figure S2, these five proteins shared conserved HPR domains, suggesting that they are good candidates for HPR. Therefore, *Cre01.g019100.t1.2*, *Cre02.g087300.t1.3*, *Cre07.g324550.t1.3*, and *Cre16.g689700.t1.3* were serially named as CrHPR2, CrHPR3, CrHPR4, and CrHPR5, respectively, according to their location in the chromosome, which, together with CrHPR1, made up the five candidate genes encoding HPR in the *Chlamydomonas* genome.

### CrHPRs Are Proteins With Hydroxypyruvate Reductase Activity

To determine how reliable the identification of potentially novel CrHPRs was and compare their enzymatic properties, the CrHPRs identified above were heterologous expressed in *E. coli* and tested different substrate and cofactor combinations (Table 1). The expression and purification of tagged-recombinant CrHPRs were assayed with SDS-PAGE gels. As shown in Supplementary Figure S3, the recombinant CrHPR proteins were obtained at high purity, and each migrated at the expected molecular weight on the gels.

**Table 1:** Activity assay of recombinant hydroxypyruvate reductases in *Chlamydomonas* (CrHPRs) with substrate and cofactor combinations.

Substrates	CrHPR1	CrHPR2	CrHPR3	CrHPR4	CrHPR5
Hydroxypyruvate:NADH	312.5 ± 19.3	2.58 ± 0.01	-	46.23 ± 1.31	1.86 ± 0.11
Hydroxypyruvate:NADPH	1.70 ± 0.24	11.03 ± 0.57	3.03 ± 0.01	-	292.40 ± 13.49
Glyoxylate:NADH	13.91 ± 0.48	2.05 ± 0.08	-	14.35 ± 0.20	0.91 ± 0.04
Glyoxylate:NADPH	0.14 ± 0.02	3.66 ± 0.09	5.65 ± 0.01	-	5.72 ± 0.19
Pyruvate:NADH	-	-	-	666.67 ± 12.36	-
Pyruvate:NADPH	-	-	-	-	-

Shown are mean activities ± SD from three independent measurements with tag-purified recombinant CrHPRs (μmol·min^−1^·mg ^−1^ protein).

With the purified recombinant CrHPRs, we measured the enzymatic activity with different substrate and cofactor combinations (Table 1). Tag-purified recombinant CrHRP1 was active in the presence of NADH and hydroxypyruvate, whereas the NADPH-dependent rate was >180 times lower (312.5 vs. 1.70 μmol·min^−1^·mg^−1^ protein). The enzyme also accepted glyoxylate as a substrate but at a considerably lower efficiency in the presence of NADH (312.5 vs. 13.91 μmol·min^−1^·mg ^−1^ protein). These parameters established CrHPR1 as the NADH-dependent HPR in *Chlamydomonas*. In contrast, CrHPR5 mainly presented as the NADPH-dependent HPR with an enzymatic activity much higher than that of any other combinations (>50 times).

CrHPR3 and CrHPR4 were detected with cofactors specificity, and they were only active in the presence of NADPH and NADH, respectively. Intriguingly, CrHPR4 could accept pyruvate as a substrate with correspondingly high enzymatic activity, suggesting its potential roles in fermentative metabolism (Burgess et al., 2015). Although it also accepted both hydroxypyruvate and glyoxylate as substrates, the catalytic efficiency is much lower than that with pyruvate (<15 times). The purified CrHPR2 showed relaxed activity with both NADH and NADPH, and hydroxypyruvate was the more favored substrate than glyoxylate.

### CrHPRs May Function in Multiple Subcellular Regions

To explore the potential functions of CrHPRs, a phylogenetic tree was generated to infer their evolution characteristics using the maximum likelihood algorithm (Kumar et al., 2015). As shown in Supplementary Figure S4, CrHPR1 was assigned into the plant subgroup, suggesting its conservation in plant-specific metabolism during evolution, and it is consistent with the alignment analysis (Supplementary Figure S1). CrHPR4 presented a close association with the photosynthetic cyanobacteria subgroup, which may imply its potential role in chloroplast considering the endosymbiotic hypothesis (Martin et al., 2015). CrHPR2, CrHPR3, and CrHPR5 likely originated from the bacteria, as they were put into the ascomycetes and firmicutes subgroups, suggesting that they may participate in mitochondrial or cytosolic metabolism.

**Figure 1:**
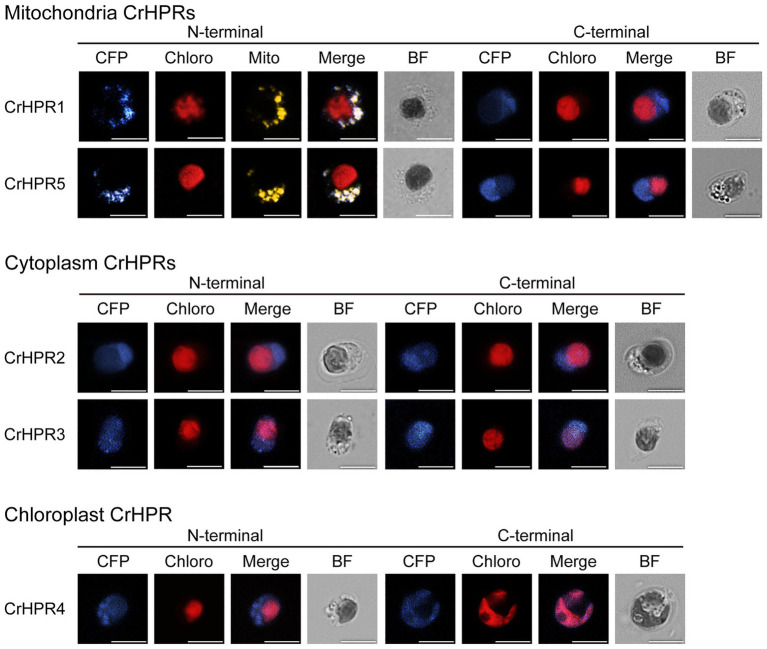
Subcellular localization of the mCerulean fluorescent protein (CFP) reporter fused with N- or C-terminal peptides from CrHPRs. Location signal at the N-terminal or C-terminal of each CrHPR is attached to the CFP reporter protein as described in the methods. Each set of images shows intact cell from the WT/CC-125 or transformants, and representative results are shown here. Mitochondria, probed with red. Mitotracker dye is shown in yellow color here to differentiate from the red autofluorescence of chloroplast. Individual imaging channels are presented. CFP, mCerulean fluorescent protein; Chloro, chlorophyll autofluorescence; Mito, MitoTracker probe; Merge, overlayed channel of CFP, Mito, and Chloro; and BF, bright field image. Scale bar, 5 μm.

To determine the reliability of subcellular localization inferred from phylogenetic analysis, either the N-terminal or the C-terminal of CrHPR was tagged with CFP as described in the Materials and Methods section. Then, the constructs were transformed into a WT/CC-125 strain, and the positive transformants were screened and assayed by laser confocal microscopy. As shown in Figure 1, CrHPR1- and CrHPR5-CFP (CFP was tagged to the C-terminal of CrHPR1 and CrHPR5) accumulated in the punctuated dots distributed in cytosol, and they likely functioned in mitochondria, as no putative peroxisomal targeting sequence, PTS1 or PTS2, was identified in their sequences (Gould et al., 1987; Swinkels et al., 1991). Furthermore, the fluorescence signals of both CrHPR1- and CrHPR5-CFP are overlapped with mitochondrion labeled with Mitotracker dye, which confirmed their mitochondria location. Using both N-terminal and C-terminal tagging only, CrHPR2 and CrHPR3 were confirmed to have diffusely presented in the cytosol. Chloroplast targeting was found for CrHPR4 supported by the overlapping between CFP-CrHPR4 signal and chlorophyll autofluorescence (Chloro; CFP was tagged to the N-terminal of CrHPR4), and it is in accordance with the previous study (Burgess et al., 2015). Therefore, speculation on the CrHPRs’ subcellular location based on phylogenetic analysis is consistent with the results from fluorescence-microscopy analysis.

### NADH-Dependent CrHPRs Take Part in Photorespiration as Major Components

Previous studies have revealed that cofactors, such as NADH and NADPH, are combined with the substrate in the catalytic reaction of HPRs (Lassalle et al., 2016). Based on this, we designed a two-step strategy to determine the CrHPRs participating in photorespiration.

**Figure 2:**
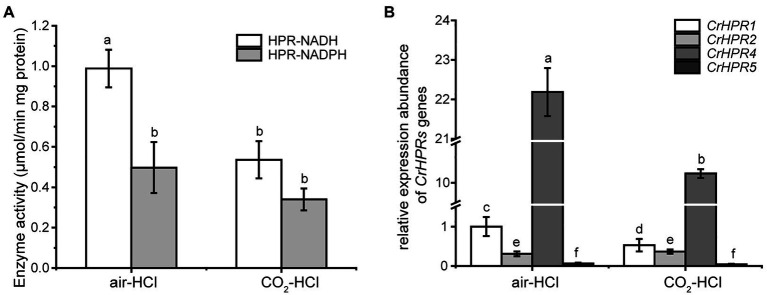
Enzyme activity assay and measurement of CrHPRs transcripts in photorespiration (air) and non-photorespiration conditions (CO_2_). **(A)** Assay of CrHPRs enzyme activity. **(B)** Detection of CrHPRs in transcriptional level. Mean values ± SD are from three independent measurements. Means denoted by the same letter did not significantly differ at *p* < 0.05.

Step 1: We examined the cofactors (NADH or NADPH) employed in photorespiration with the cell extracts, from which we could infer the related CrHPRs on the basis of their catalytic characteristics (Table 1). The detected enzymatic activity is from all of the NADH- or NADPH-dependent CrHPRs. As shown in Figure 2A, the enzyme activity of NADH-dependent HPRs was much higher in photorespiration induced by air condition than that under non-photorespiration condition (CO_2_ condition), while the activity of NADPH-dependent HPRs was only slightly changed, which demonstrated the major roles of NADH-dependent CrHPRs in photorespiration. Since CrHPR1, CrHPR2, CrHPR4, and CrHPR5 were detected with the activity of HPRs in the presence of NADH (Table 1), thus, their participation in photorespiration was examined further.
Step 2: If CrHPRs function in photorespiration, they could be induced in such conditions. Based on the assumption, we monitored their transcriptional profiles to determine the CrHPRs involved in photorespiration. As indicated in Figure 2B, a 2-fold increase in CrHPR1 and CrHPR4 was detected in their transcriptional levels when cells were transferred from CO_2_ to air conditions, suggesting their potential roles in photorespiration. In contrast, we did not observe a significant difference in CrHPR2 or CrHPR5 transcripts between CO_2_ and air conditions, suggesting their fewer dominant roles in photorespiration compared with CrHPR1 or CrHPR4 (Figure 2B).

Together, the NADH-dependent CrHPRs take part in photorespiration as major components, in which CrHPR1 and CrHPR4 may play more important roles than CrHPR2 and CrHPR5 under the tested conditions.

### Knockout of CrHPR1 Impairs Photorespiration

**Figure 3:**
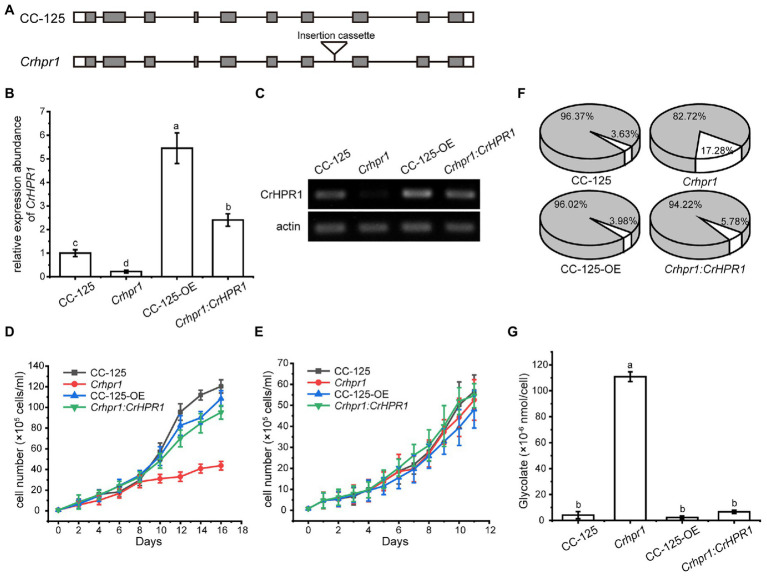
Knockout of CrHPR1 impairs photorespiration. **(A)** Schematics of CrHPR1 structures in WT/CC-125 and *Crhpr1*. **(B)** qRT-PCR analysis of CrHPR1 transcripts in the strains. **(C)** RT-PCR of analysis of CrHPR1 transcripts in the strains. Growth curve of strains in **(D)** air and **(E)** 3% CO_2_ condition. **(F)** The ratio of oxidation and carboxylation reaction of ribulose bisphosphate carboxylase/oxygenase (Rubisco) in air. Carboxylation reaction: white sector. Oxidation reaction: gray sector. **(G)** Concentration of glycolate detected in the medium of each strain. Mean values ± SD presents data from three measurements. Means denoted by the same letter did not significantly differ at *p* < 0.05.

CrHPR1 was confirmed with high NADH-dependent activity and may play a major role in photorespiration (Table 1; Figure 2). To explore its physiological function, we set out to isolate the insertion strain for *CrHPR1*/*Cre06.g295450.t1.2* within the mutant library generated previously (Cheng et al., 2017). As shown in Figure 3A, the paromomycin resistance cassette *AphVIII* was inserted into the seventh intron in *Crhpr1*, which was confirmed by genome sequencing (data not shown). The knockout was then verified by qRT-PCR analysis using primers that span the cassette insertion site as described in the Materials and Methods section. The mature transcript was found to be greatly disrupted in the mutant as indicated in Figures 3B,C. These results confirmed the knockout of *CrHPR1/Cre06.g295450.t1.2* in the insertion line *Crhpr1*, and it was used for further study.

As shown in Figure 3E, *Crhpr1* presented no noticeable difference in growth to WT when they were cultured in tris-minimal medium under high CO_2_ conditions. However, photorespiratory defects of *Crhpr1*, such as retarded growth and decreased ratio of Rubisco (oxidation/carboxylation), were observed when the cells were transferred to the air condition (Figures 3D,F; Supplementary Table S3). Interestingly, a stronger ability to process photosynthetic electron flow was detected in *Crhpr1*, manifesting as the elevation in the maximum photochemical quantum yield of PSII, maximum electron transfer efficiency, and reduction in minimum saturating irradiance (Supplementary Figure S5). The phenotypic defects of *Crhpr1* may result from the leakage of photosynthetically fixed carbon, which is supported by more glycolate excreted into the medium by *Crhpr1* as measured in Figure 3G. Overexpression of CrHPR1 in WT/CC-125 did not bring visually phenotypic changes (Figures 3D–G).

The phenotypes of *Crhpr1* detected above were restored to the WT/CC-125 level in the rescued strain *Crhpr1:CrHPR1* (Figures 3B–G; Supplementary Figure S5), which provided evidence that the photorespiration is disrupted in *Crhpr1* and, thus, that the function of CrHPR1 is closely related to photorespiration.

### CrHPR2 Knockdown Strains Show Photorespiratory Defects at *CrHPR1* Background

**Figure 4:**
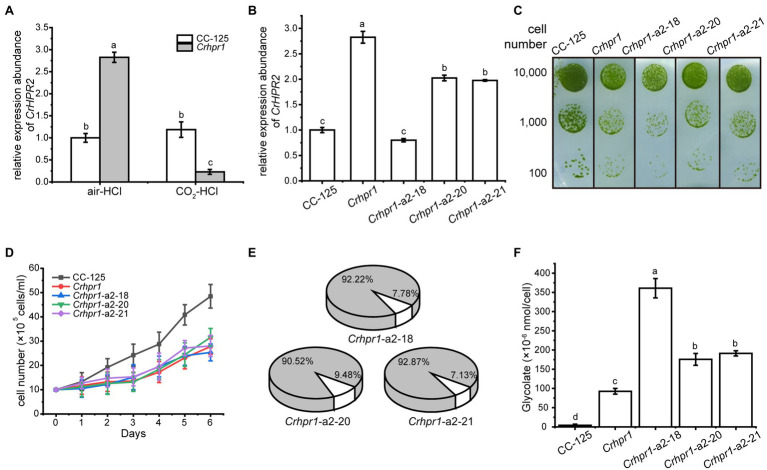
CrHPR2 knockdown strains show photorespiratory defects at *Crhpr1* background. **(A)** Measurement of CrHPR2 transcripts at both WT/CC-125 and *Crhpr1* background in air condition. **(B)** Measurement of CrHPR2 transcripts in *Crhpr1* and *Crhpr1-a2* strains. **(C)** Spot tests showing growth of *Crhpr1* and *Crhpr1-a2* strains. **(D)** Growth curves of *Crhpr1* and *Crhpr1-a2* strains in air. **(E)** The ratio of oxidation and carboxylation reaction of Rubisco in *Crhpr1-a2* strains. Carboxylation reaction: white sector. Oxidation reaction: gray sector. **(F)** Concentration of glycolate detected in the medium of each strain. Mean values ± SD presents data from three measurements. Means denoted by the same letter did not significantly differ at *p* < 0.05.

Although CrHPR2 was detected with NADPH-dependent HPR activity, it was not induced in WT/CC-125 under photorespiratory conditions as indicated by the qRT-PCR analysis (Figure 2B), and no difference in phenotypes of CrHPR2 knockdown strains and WT/CC-125 was observed (Supplementary Table S3; Supplementary Figure S6). We speculated that CrHPR2 performs the compensatory functions of CrHPR1, and it may act as the major component in the extra-mitochondria hydroxypyruvate-reducing pathway in *Chlamydomonas*, which could catalyze the redundant hydroxypyruvate that penetrated from mitochondria. To test this possibility, we compared the transcript level of CrHPR2 in WT/CC-125 and *Crhpr1*. As shown in Figure 4A, the expression of CrHPR2 was greatly induced in the air when CrHPR1 was deleted, which verified the potential role of CrHPR2 in the photorespiration mentioned above. To explore its physiological function, we generated the CrHPR2 knockdown strains at the *Crhpr1* background, yielding serial *Crhpr1-a2* mutants. The qRT-PCR analysis indicated that the CrHPR2 transcript in *Crhpr1-a2* strains was reduced to various extents of that in WT/CC-125 (Figure 4B), which confirmed the disruption of the CrHPR2 expression. Thus, they were used for further analysis.

Although the expression of CrHPR2 was greatly destroyed, no visible difference in growth was observed between *Crhpr1* and *Crhpr1-a2* strains when the cells were cultured in air, regardless of the solid or liquid medium (Figures 4C,D). Nevertheless, the catalytic rate of Rubisco (carboxylation/oxidation) of *Crhpr1-a2* strains was much lower than that of *Crhpr1*, especially that the carboxylation rate was greatly affected in *Crhpr1-a2* (Figure 4E; Supplementary Table S3). These results suggest that the photosynthetic activity of *Crhpr1-a2* may have been affected by the increased glycolate excreted by the strains. We further measured the concentration of glycolate secreted to the medium by respective mutants, and the results indicated that more glycolate was indeed released by *Crhphr1-a2* strains than those of *Crhpr1* (Figure 4F).

This evidence confirms that photorespiration is affected in the CrHPR2 knockdown strains, and CrHPR2 is indeed associated with photorespiration. This exacerbated excretion of glycolate by *Crhpr1-a2* may bring damage to photosynthesis to some extent, manifesting as decreased quantum yield efficiency in PSII, electron transfer rate, and minimum suturing irradiance (Supplementary Figure S7).

### CrHPR4 Participates in Photorespiration as a Chloroplast-Targeting Glyoxylate Reductase

**Figure 5:**
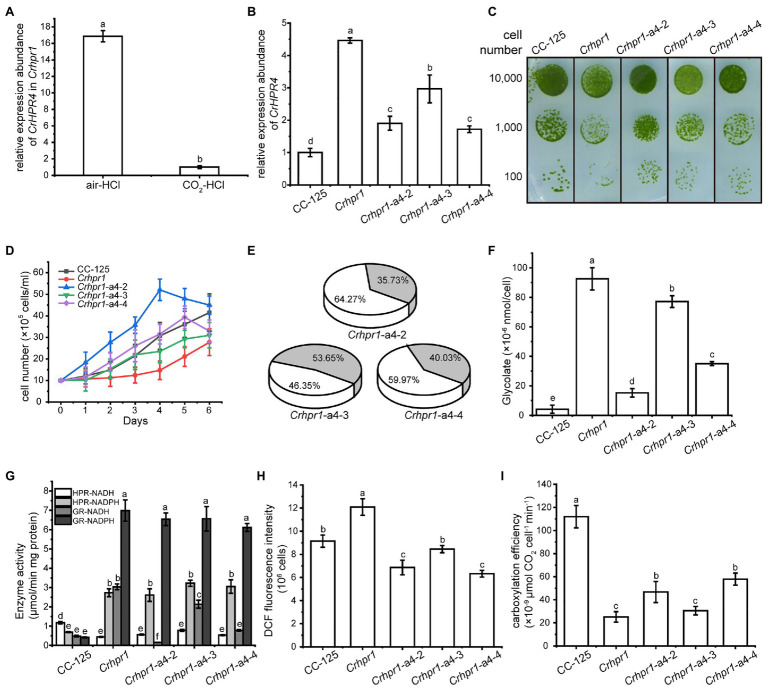
CrHPR4 participates in photorespiration as a chloroplast-targeting glyoxylate reductase. **(A)** Measurement of CrHPR4 transcripts at both WT/CC-125 and *Crhpr1* background in air condition. **(B)** Measurement of CrHPR4 transcripts in *Crhpr1* and *Crhpr1-a2* strains. **(C)** Spot tests showing growth of *Crhpr1* and *Crhpr1-a4* strains. **(D)** Growth curves of *Crhpr1* and *Crhpr1-a4* strains. **(E)** The ratio of oxidation and carboxylation reaction of Rubisco in *Crhpr1-a4* strains. Carboxylation reaction: white sector. Oxidation reaction: gray sector. **(F)** Concentration of glycolate detected in the medium of each strain. **(G)** Enzyme activity assay of *Crhpr1* and *Crhpr1-a4* strains. HPR-NADH: white bars. HPR-NADPH: light gray bars. GR-NADH: gray bars. GR-NADPH: dark gray bars. **(H)** Determination of reactive oxygen species (ROS) in *Crhpr1* and *Crhpr1-a4* strains treated with salicylhydroxamic acid. **(I)** Determination of carboxylation efficiency in *Crhpr1* and *Crhpr1-a4* strains treated with salicylhydroxamic acid. Mean values ± SD from three measurements. Means denoted by the same letter did not significantly differ at *p* < 0.05.

CrHPR4 was greatly induced in both WT/CC-125 and *Crhpr1* strains in the air (Figures 2B, 5A), demonstrating its participation in photorespiration. To uncover its detailed function in photorespiratory physiology, we generated *CrHPR4* knockdown strains at WT/CC-125 background, yielding *WT/CC-125-a4*. Both photosynthetic activity and growth of *WT/CC-125-a4* strains were only slightly affected (Supplementary Figure S8), which is consistent with the previous study (Burgess et al., 2015). However, the ratio of Rubisco (oxidation/carboxylation) was detected with a lower value in *WT/CC-125-a4* strains than that in WT/CC-125 (Supplementary Table S8), suggesting the relationship of CrHPR4 with photorespiration to some extent. To further explore the possibility, we generated CrHPR4 knockdown strains at *Crhpr1* background, yielding *Crhpr1-a4*. As determined by the qRT-PCR analysis, a reduced expression level of CrHPR4 was detected in *Crhpr1-a4* strains (Figure 5B), and they were used in the following studies.

Apparently, visually enhanced growth was observed for *Crhpr1-a4* strains compared with that of *Crhpr1* in both the solid and liquid media (Figures 5C,D). The highly accumulated biomass of *Crhpr1-a4* possibly resulted from its efficient activity in both photosynthesis and CO_2_ fixation (Figure 5E; Supplementary Figure S10; Supplementary Table S3). Unexpectedly, less glycolate, intermediate in photorespiration, was excreted by *Crhpr1-a4* compared with both *Crhpr1* and *Crhpr1-a2* (Figure 5F). This prompted us to investigate whether CrHPR4 still performs its functions as an HPR. We then examined the enzymatic activity with cell extracts from respective strains and determined the difference (Figure 5G). Intriguingly, the enzymatic activity of NADH-dependent glyoxylate reductase, rather than HPR, was significantly impaired/decreased in *Crhpr1-a4* strains compared with that in *Crhpr1* (Figure 5G). This evidence established the relationship of CrHPR4 with photorespiration, but it may mainly function as glyoxylate reductase and not HPR, and its physiological effects seem to be different from those of CrHPR1 and CrHPR2.

Considering that CrHPR4 was targeted to the chloroplast (Figure 1), it likely plays role in the glycolate-quinone oxidoreductase system, which could be inhibited by salicylhydroxamic acid (SHAM; Goyal, 2002). To test this possibility, we added SHAM to the medium and found that the excretion of glycolate was indeed suppressed in *Crhpr1-a4* strains (Figure 5F). Moreover, the halted glycolate-quinone oxidoreductase reaction with SHAM resulted in less reactive oxygen species, which is the byproduct of the oxidoreductase system (Figure 5H) and higher carboxylation efficiency (Figure 5I).

Together, these results provide evidence that CrHPR4 may take part in photorespiration by acting as the chloroplasdial glyoxylate reductase in the glycolate-quinone oxidoreductase system, which differentiates it from CrHPR1 and CrHPR2.

## Discussion

### The CrHPR Proteins Identified Here Are Previously Uncharacterized Photorespiratory Components With Hydroxypyruvate Reductase

In an effort to identify all the HPRS in the *Chlamydomonas* genome, we initially used the BLAST function with the protein sequence of CrHPR1 as a reference to search against the *Chlamydomonas* proteome. The analysis generated a list of five candidate novel CrHPRs (including CrHPR1 itself), in which the conserved domains of HPR, such as NAD(P)-binding motif, NAD recognition sites, substrate-orienting, and catalytic pair domains, were identified (Supplementary Figures S1, S2). Further study of the five CrHPRs by enzymatic assay showed that all five are indeed detected with the activity of HPR (Table 1). Subcellular localization (Figure 1) and phenotypic characterization of respective mutants (Figures 3–5) provide evidence that some of these candidate proteins, in fact, act in photorespiration. Thus, we have globally identified the CrHPRs and explored their functions that were previously unknown.

### CrHPRs Present Distinct Subcellular Location Patterns and Coordinately Function in Photorespiration

Compared with the higher plants, photorespiration was assumed to pass through mitochondria rather than peroxisome in *Chlamydomonas* (Nakamura et al., 2005), which is supported by the detected mitochondrial CrHPR activity (Stabenau, 1974). Husic and Tolbert (1987) further demonstrated that NADH-dependent CrHPRs may function as major components of HPRs in photorespiration but not yet known at the sequence level. Here, we identified two mitochondrion-targeting CrHPRs, CrHPR1, and CrHPR5, assayed with NADH-dependent activity for the first time according to our knowledge (Figure 1; Table 1), and they are likely the proteins previously shown to be associated with photorespiration (Husic and Tolbert, 1987). Especially CrHPR1, which was experimentally verified as having high NADH-dependent enzymatic activity, may contribute to most of the HPR activities measured by Husic and Tolbert (1987). Therefore, it may not be coincidental that *Crhpr1* developed photorespiratory defects in the air (Figures 3D–G). CrHPR5, active in the presence of NADPH, may have a function related to the glutathione peroxidase or peroxiredoxin antioxidant systems that need plenty of NADPH converted from NADH.

However, Husic and Tolbert (1987) did not know that there are bypass pathways of photorespiration, a conclusion that is supported by both previous and present studies (Timm et al., 2008, 2011). In this study, we found that CrHPR2 and CrHPR4, which are proved to be related to photorespiration (Figures 4, 5), were targeted to cytosol and chloroplast (Figure 1), respectively. They may function as such components of the bypass pathway of photorespiration, and it will be of interest to determine their detailed functions as discussed in the below sections.

### CrHPR1 Acts as the Major NADH-Dependent HPR in *Chlamydomonas* and Functions Differently From HPR1 in Higher Plants

CrHPR1 was assigned into the plant-specific subgroup in the phylogenetics analysis (Supplementary Figure S4), and it has been well inherited through evolution implicated by the presence of homologs in higher plants (Supplementary Figure S1). However, the function of CrHPR1 in photorespiration may be different from its homologs in higher plants according to this study.

**Figure 6:**
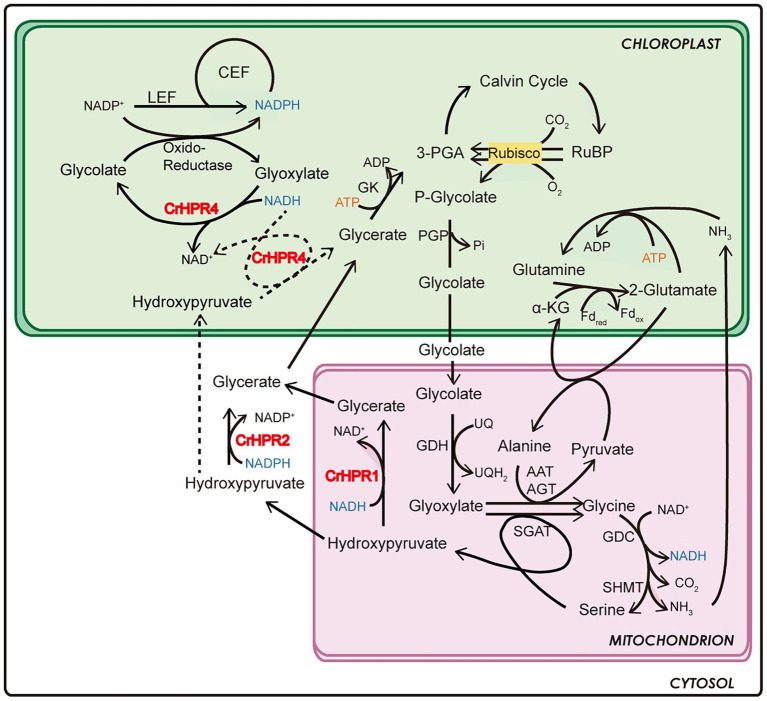
Schematics of the functional mechanism of CrHPRs in photosynthesis and photorespiration based on this study.

Unlike the peroxisome-location of HPR1 in higher plants, CrHPR1 was targeted to mitochondria (Figure 1), which supports the assumption that photorespiration may pass through mitochondria rather than peroxisome in *Chlamydomonas* (Nakamura et al., 2005). Further investigation revealed that *Crhpr1* presented obvious photorespiratory defects and retarded growth in the air (Figure 3), which are greatly different from the no visually noticeable phenotypes of *hpr1* mutants in high plants (Timm et al., 2008; Cousins et al., 2011). This leads us to suggest that CrHPR1 acts as the major NADH-dependent HPR in *Chlamydomonas* (Figure 6), and the role of CrHPR1 in photorespiration seems to be much more dominant than that of HPR1 in higher plants. Apparently, the cytosolic or chloroplast bypass pathway of photorespiration (Figures 4, 5), performed by CrHPR2 and CrHPR4, could not fully compensate for the lost function of mitochondrial CrHPR1. Therefore, the novel phenotypes of *Crhpr1* described here expand the range of HPR phenotypes associated with photorespiratory defects. Interestingly, the overexpression of CrHPR1 did not bring a great phenotypic change, although the transcript level was detected much higher in CC-125-OE strains. We speculate that the CrHPR1 activity may be regulated at the post transcriptional level or that the phenotypic change occurs in a time frame not captured by induction time point. We could not rule out the possibility that CrHPR1 may function under stress conditions, and then the phenotype of CrHPR1 overexpression strains could be obviously developed. Thus, the underlying roles of CrHPR1 under various conditions remain to be globally explored.

During the course of the analysis, we also noticed that *Chlamydomonas* and high plants respond differently to photorespiratory defects. In *Arabidopsis*, 2-phosphoglycolate is accumulated and metabolized by the G6P shunt in the *hpr1* mutant (Li et al., 2019), and hydroxypyruvate is converted into glycolate after decarboxylation and oxidation, then glycolate reenters the core photorespiration pathway (Missihoun and Kotchoni, 2018). However, the *Crhpr1* mutant excretes a large amount of glycolate to balance the internal environment (Figure 3G), which resulted in the greatly reduced efficiency of carbon fixation. It is likely that more complex photorespiration and adaption mechanisms have been adopted during evolution, but the underlying cause of the difference between *Chlamydomonas* and higher plants remains to be clarified.

### CrHPR2 Participates in the Cytosolic Bypass of Photorespiration

The general assumption that the conversion of hydroxypyruvate to glycerate is exclusively performed by the mitochondria- or peroxisome-targeted HPR1 may not be comprehensive (Timm et al., 2008, 2011; Ye et al., 2014; Missihoun and Kotchoni, 2018), considering that HPR2 could participate in the cytosolic bypass of photorespiration in both *Chlamydomonas* and higher plants (Table 1; Figure 4; Timm et al., 2008). The determination of the functions of CrHPR2 in photorespiration is based on evidence from several aspects as follows.

In the initial analysis, we found that CrHPR2 was assigned into the bacterial subgroup, which suggests that its participation may not be limited to light-related metabolism (Supplementary Figure S4). To further investigate its function, we generated the CrHPR2 knockdown strains at WT/CC-125 background, but no obvious photorespiratory defect was observed (Supplementary Figure S6). When CrHPR2 was knocked down in *Crhpr1*, more severe photorespiratory defects of *Crhpr1-a2* could be detected than those of *Crhpr1* (Figure 4). This evidence implies the participation of CrHPR2 in photorespiration, but it may play a compensatory role to some extent, which is supported by the result from the qRT-PCR analysis (Figures 4A, 6). If so, the evidence mentioned above supports the permeability of the mitochondria matrix for hydroxypyruvate. Thus, the data provide indirect evidence that hydroxypyruvate could easily equilibrate with cytosol when no CrHPR1 is present within the mitochondria. Despite the presence of mitochondria channeling of hydroxypyruvte remains unclear (Keech et al., 2017), it is not unlikely that such equilibration mentioned above occurs in WT/CC-125.

Last but not least, it is of importance to build the interaction network of cytosolic CrHPR2, which would provide more details on CrHPR2 in both photorespiration and other metabolic pathways. In addition, CrHPR3 was also localized to cytosol but did not show a function similar to that of CrHPR2. How CrHPR2 and CrHPR3 coordinate with each other in the cytosolic bypass of photorespiration remains to be explored.

### CrHPR4 Targeted to the Chloroplast, Mainly Plays Roles in Photorespiration as Glyoxylate Reductase Within This Compartment

Here, we report to our knowledge a previously uncharacterized enzyme that could reduce hydroxypyruvte, glyoxylate, and pyruvate (Table 1), and hence could directly or indirectly contribute to photorespiration in *Chlamydomonas*. Considering its multiple substrates and preference for the cofactor NADH, the identified CrHPR4 could support mitochondrial and cytosolic CrHPRs as well as chloroplastidial and cytosolic glyoxylate reductases (Ching et al., 2012; Brikis et al., 2017) for an optimal reduction of the respective intermediates (Figure 2). Therefore, the observed effects of CrHPR4 on photorespiration clearly suggest its involvement in this process (Figure 5).

By combining the data of enzymatic characteristics and the analysis of *Crhpr1-a4* strains, we proposed the detailed mechanism for CrHPR4 participating in photorespiration as shown in Figure 6: CrHPR4 was detected with the activity of glyoxylate reductase (Table 1), and it may act in the glycolate-quinone oxidoreductase system, which is supported by the results presented in Figure 5. When CrHPR4 is knocked down in *Crhpr1*, excess glyoxylate is converted into CO_2_, which directly inhibits the oxidation reaction of Rubisco, while promoting the carboxylation reaction (Figure 5E; Supplementary Table S3). Meanwhile, the production of glycolate mediated by CrHPR4 is greatly disrupted in *Crhpr1-a4* strains, resulting in less excretion of glycolate into the medium (Figure 5F). Together, the knockdown of CrHPR4 results in increased CO_2_ fixation and less loss in photosynthetic fixed carbon (Figures 5H,I). As a result, *Crhpr1-a4* was observed with more robust growth compared with *Crhpr1* (Figures 5C,D). Considering the great effects of CrHPR4 on the conversion of glyoxylate into glycolate, it may function as the dominant glyoxylate reductase in the chloroplast. It is undeniable that CrHPR4 has linked photosynthesis and photorespiration closely *via* the glycolate metabolism, and the fine-tuning mechanism remains unidentified yet.

Interestingly, CrHPR4 was detected with the activity of pyruvate reductase, which is consistent with the previous study (Burgess et al., 2015). As pyruvate reductase, CrHPR4, however, may mainly play an important role in anaerobic/fermentation metabolism (Burgess et al., 2015). It seems likely that CrHPR4 could participate in both photorespiration and anaerobic metabolism by acting as a bifunction enzyme, but how CrHPR4 performs roles in two pathways remains to be identified. Thus, the investigation of the interaction network of CrHPR4 in chloroplast could provide more details. Moreover, CrHPR4 was also detected with the activity of HPR, but how hydroxypyruvate is transported from mitochondrion to chloroplast is not identified yet (Reumann and Weber, 2006; Keech et al., 2017). Thus, it would be very informative to analyze and compare the “-omics” of *Crhpr1-a4* under photorespiratory and non-photorespiratory conditions as fermentation to uncover its elusive functions and exact physiological role.

In conclusion, although the role of CrHPR4 will need further investigation, this study presents strong indications that the enzyme is closely associated with the photorespiratory process and could at least partially participate in chloroplast glycolate metabolism. Moreover, CrHPR4 could display a possible link of photorespiration to photosynthesis and fermentation that remains to be identified. It is particularly important to clarify the underlying mechanism of CrHPR4 functioning in multiple pathways, and it will benefit both the construction of plant metabolic network and crop improvement by synthetic biology.

## Concluding Remarks

The results reveal that the CrHPRs are far more complex than previously recognized, and provide a greatly expanded knowledge base to understand their functions in photorespiration for future study. Considering the presence of multiple CrHPRs in the *Chlamydomonas* genome and phenotypes of the mutants, it will be of great interest to uncover the detailed mechanism of how they coordinate with each other when performing roles in photorespiration.

Hydroxypyruvate reductases in *Chlamydomonas* could link photorespiration to photosynthesis and fermentation, and they may act as the central hub in the coordination of metabolism. Hence, the studies here will benefit the construction of plant metabolic network and provide important clues for crop improvement by genetic engineering. Meanwhile, glycolate could be converted into methane (Günther et al., 2012), and it will be of interest to explore the potentiality of converting glycolate into bioenergy.

## Data Availability Statement

The original contributions presented in the study are included in the article/**Supplementary Material**, further inquiries can be directed to the corresponding authors.

## Author Contributions

MS, LZ, and YW conceived the project, analyzed the data, and wrote and revised the manuscript. MS performed the experiments. All authors contributed to the article and approved the submitted version.

### Conflict of Interest

The authors declare that the research was conducted in the absence of any commercial or financial relationships that could be construed as a potential conflict of interest.

## Footnotes

^1^https://www.chlamycollection.org

^2^https://www.ncbi.nlm.nih.gov/tools/primer-blast/index.cgi?LINK_LOC=BlastHome

^3^http://wmd3.weigelworld.org

^4^https://www.ebi.ac.uk/Tools/hmmer/search/phmmer

^5^http://algaepath.itps.ncku.edu.tw/algae_path/home.html
