# Supplementary material for: Identification and Characterization of Genes Encoding the Hydroxypyruvate Reductases in Chlamydomonas Reveal Their Distinct Roles in Photorespiration
Source: Front Plant Sci. 2021 Jun 24;12:690296. doi: 10.3389/fpls.2021.690296 (PMC8264790; doi:10.3389/fpls.2021.690296)
Supplement: Supplementary file 1 [file Data_Sheet_1.docx]

**Supplementary Tables**

**Table S1.** All Chlamydomonas strains used in the work

| Strains | Description |
| --- | --- |
| CC125 | Wild type |
| *Crhpr1* | CrHPR1 insertion mutant |
| CC125-O | CrHPR1 overexpression strain |
| *Crhpr1*-O | Rescued strain of *Crhpr1* |
| CrHPR1-N | Strain containing N-terminal target signal of CrHPR1 with CFP |
| CrHPR1-C | Strain containing C-terminal target signal of CrHPR1 with CFP |
| CrHPR2-N | Strain containing N-terminal target signal of CrHPR2 with CFP |
| CrHPR2-C | Strain containing C-terminal target signal of CrHPR2 with CFP |
| CrHPR3-N | Strain containing N-terminal target signal of CrHPR3 with CFP |
| CrHPR3-C | Strain containing C-terminal target signal of CrHPR3 with CFP |
| CrHPR4-N | Strain containing N-terminal target signal of CrHPR4 with CFP |
| CrHPR4-C | Strain containing C-terminal target signal of CrHPR4 with CFP |
| CrHPR5-N | Strain containing N-terminal target signal of CrHPR5 with CFP |
| CrHPR5-C | Strain containing C-terminal target signal of CrHPR5 with CFP |
| *Crhpr1*-a2-18 | CrHPR2 knockdown strains at *Crhpr1* background |
| *Crhpr1*-a2-20 | CrHPR2 knockdown strains at *Crhpr1* background |
| *Crhpr1*-a2-21 | CrHPR2 knockdown strains at *Crhpr1* background |
| *Crhpr1*-a4-2 | CrHPR4 knockdown strains at *Crhpr1* background |
| *Crhpr1*-a4-3 | CrHPR4 knockdown strains at *Crhpr1* background |
| *Crhpr1*-a4-4 | CrHPR4 knockdown strains at *Crhpr1* background |
| CC-125-a2-3 | CrHPR2 knockdown strains at CC-125 background |
| CC-125-a2-5 | CrHPR2 knockdown strains at CC-125 background |
| CC-125-a4-19 | CrHPR4 knockdown strains at CC-125 background |
| CC-125-a4-20 | CrHPR4 knockdown strains at CC-125 background |

**Table S2.** Oligonucleotides used in this work

| Name | Sequence (5’-3’) |
| --- | --- |
| CrHPR1-qRT-F | CGCGTGGAGACGGTGGAGGAGGT |
| CrHPR1-qRT-R | ACCAGAACCGCCGTCGGCTTCAT |
| CrHPR2-qRT-F | ACGTGACGACCCGCTGTACT |
| CrHPR2-qRT-R | GCGTGATGTTCTCGCACAGG |
| CrHPR3-qRT-F | TGGGCTATGGTGTCATGGGC |
| CrHPR3-qRT-R | ACGACTCACGACCCGAGAAC |
| CrHPR4-qRT-F | GGACCCCCACCGGACTAAAC |
| CrHPR4-qRT-R | AGTCTCACAACAGGACCGCC |
| CrHPR5-qRT-F | GCCGCGTGAGAGGATGTACT |
| CrHPR5-qRT-R | GCGCCCACCATTTTGTCAGT |
| actin-F | GACCCCGTTCCCCCGATCTA |
| actin-R | AGCAAAGCCAGCCTTCACCA |
| CrHPR1-pro-F | CCATGGCGCTCGCCCGCGC |
| CrHPR1-pro-R | CTCGAGCTCCGCCACGGTCTTAAGCTTG |
| CrHPR2-pro-F | CCATGGCAGCCGCGAGCGC |
| CrHPR2-pro-R | CTCGAGGCACAGCCGGTGCAGCAGC |
| CrHPR3-pro-F | CATATGGCAAGTGCGTCTGCCC |
| CrHPR3-pro-R | CTCGAGATACCCGACTGACTTGTTCGC |
| CrHPR4-pro-F | CATATGATGCTATTATCATCCACCCAGA |
| CrHPR4-pro-R | CTCGAGGGTCTTGCCGGCGGGGG |
| CrHPR5-pro-F | CATATGCTGCTCCAGGGCAAAAC |
| CrHPR5-pro-R | CTCGAGGTAGCCGTTCCGCTTGTCGC |
| CrHPR1-O-F | CTCGAGATGGCGCTCGCCCGCGC |
| CrHPR1-O-R | GAATTCCTACTCCGCCACGGTCTTAAGC |
| CrHPR1-NT-F | CTCGAGGCGCTCGCCCGCGCGTTC |
| CrHPR1-NT-R | GGATCCCTCCGCCACGGTCTTAAGCTTG |
| CrHPR1-CT-F | GGATCCGCGCTCGCCCGCGCGTTC |
| CrHPR1-CT-R | GAATTCCTACTCCGCCACGGTCTTAAGC |
| CrHPR2- NT-F | CTCGAGGCAGCCGCGAGCGCTGTAAG |
| CrHPR2- NT-R | GGATCCGCACAGCCGGTGCAGCAGC |
| CrHPR2-CT-F | GGATCCGCAGCCGCGAGCGCTGTAAG |
| CrHPR2-CT-R | GAATTCTCAGCACAGCCGGTGCAGC |
| CrHPR3- NT-F | CTCGAGGCAAGTGCGTCTGCCCAGT |
| CrHPR3- NT-R | GGATCCATACCCGACTGACTTGTTCGC |
| CrHPR3-CT-F | GGATCCGCAAGTGCGTCTGCCCAGTT |
| CrHPR3-CT-R | GAATTCCTAATACCCGACTGACTTGTTCGC |
| CrHPR4- NT-F | CTCGAGATGCTATTATCATCCACCCAGAGC |
| CrHPR4- NT-R | GGATCCGGTCTTGCCGGCGGGGGC |
| CrHPR4-CT-F | GGATCCATGCTATTATCATCCACCCAGAGC |
| CrHPR4-CT-R | GAATTCTTAGGTCTTGCCGGCGGG |
| CrHPR5- NT-F | CTCGAGCTGCTCCAGGGCAAAACG |
| CrHPR5- NT-R | GGATCCGTAGCCGTTCCGCTTGTCG |
| CrHPR5-CT-F | GGATCCCTGCTCCAGGGCAAAACG |
| CrHPR5-CT-R | GAATTCTCAGTAGCCGTTCCGCTTGT |
| amiRNA-CrHPR2 | CCGCTAATGATGATCCACATAtctcgctgatcggcaccatgggggtggtggtgatcagcgctaTATGAGGATCATCATTAGCGG |
| amiRNA-CrHPR4 | TTTGATGTCATAGGCGGACACtctcgctgatcggcaccatgggggtggtggtgatcagcgctaGTGTTTGCCTATGACATCAAA |

F, forward; R, reverse. Underlined letters indicate restriction sites.

**Table S3.** Oxidation/carboxylation rate of Rubisco of different strains in air

|  | Jc(×10^-9^μmol cell^-1^ min^-1^) | Jo(×10^-9^μmol cell^-1^ min^-1^) | Jo/Jc ratio |
| --- | --- | --- | --- |
| CC-125 | 1.01±0.09 | 26.84±0.33 | 26.39±2.20 |
| *Crhpr1* | 6.45±0.72 | 30.87±0.49 | 5.87±0.57 |
| CC-125-OE | 1.19±0.13 | 28.69±0.34 | 24.11±3.28 |
| *Crhpr1:CrHPR1* | 1.58±0.13 | 25.74±0.22 | 16.29±1.61 |
| CC-125*-*a2-3 | 1.18±0.07 | 27.43±0.43 | 23.30±3.50 |
| CC-125*-*a2-5 | 0.84±0.03 | 22.56±0.56 | 26.91±3.26 |
| *Crhpr1*-a2-18 | 2.24±0.05 | 26.56±0.73 | 11.86±0.60 |
| *Crhpr1*-a2-20 | 3.56±0.17 | 33.98±0.96 | 9.54±0.76 |
| *Crhpr1*-a2-21 | 2.25±0.18 | 29.32±0.37 | 13.03±1.31 |
| CC-125*-*a4-19 | 3.55±0.13 | 29.02±0.72 | 8.16±1.00 |
| CC-125*-*a4-20 | 4.02±0.28 | 31.08±0.97 | 7.73±1.57 |
| *Crhpr1*-a4-2 | 13.56±0.73 | 7.54±0.13 | 0.56±0.04 |
| *Crhpr1*-a4-3 | 11.23±0.15 | 13.00±0.08 | 1.16±0.02 |
| *Crhpr1*-a4-4 | 13.23±0.27 | 8.83±0.14 | 0.67±0.02 |

Mean activities ±SD from three measurements are shown.

**Supplementary Figures**


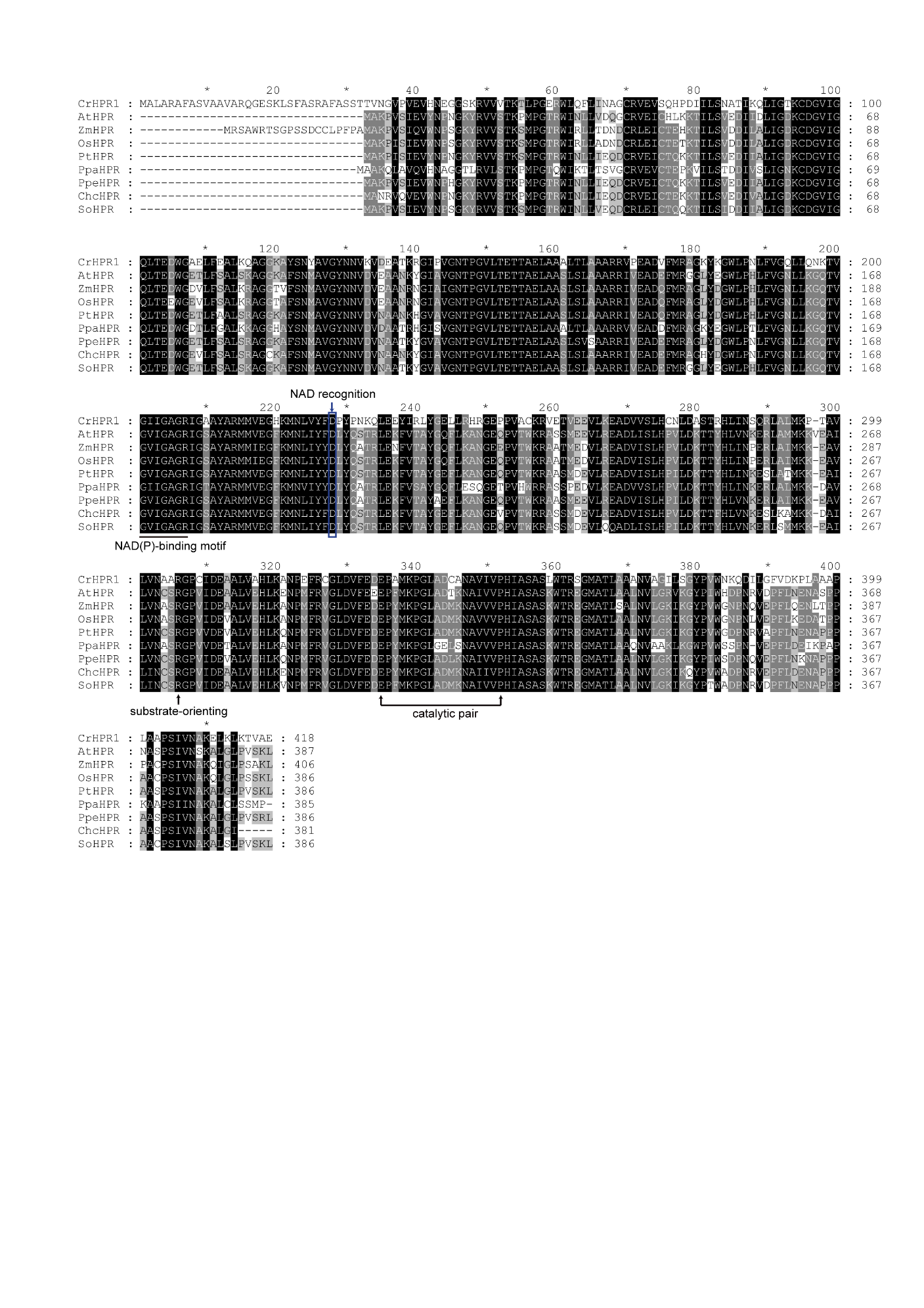


**Fig. S1.** **Sequence alignment of hydroxypyruvate reductase homologues from select species.**

Black highlighting indicates identical residues; Light gray highlighting indicates conserved residues with a value of above 60%. Gray highlighting indicates conserved residues with a value of above 80 %. Gaps introduced to optimize sequence alignment are indicated by hyphen. The number to the right of each protein sequence indicates the number of amino acids in the protein. The conserved domain, including NAD-recognition, NAD-binding motif, substrate orienting and catalytic pair, are indicated with blue rectangle box. Amino acid sequences are: AtHPR: hydroxypyruvate reductase of *Arabidopsis thaliana* (Accession No. AT1G68010); ChcHPR: hydroxypyruvate reductase of *Cucurbita hybrid cultivar* (Accession No. BAA08411); CrHPR1: hydroxypyruvate reductase 1 of *Chlamydomonas reinhardtii* (Accession No. Cre06.g295450.t1.2); OsHPR: hydroxypyruvate reductase of *Oryza sativa* (Accession No. LOC_Os02g01150); PpaHPR: hydroxypyruvate reductase of *Physcomitrella patens* (Accession No. Pp3c18_12500); PpeHPR: hydroxypyruvate reductase of *Prunus persica* (Accession No. Prupe.8G166000); PtHPR: hydroxypyruvate reductase of *Populus trichocarpa* (Accession No. Potri.004G175800); SoHPR: hydroxypyruvate reductase of *Spinacia oleracea* (Accession No. XP_021851981); ZmHPR: hydroxypyruvate reductase of *Zea mays* (Accession No. GRMZM2G074282); The sequences are aligned using ClustalW software.


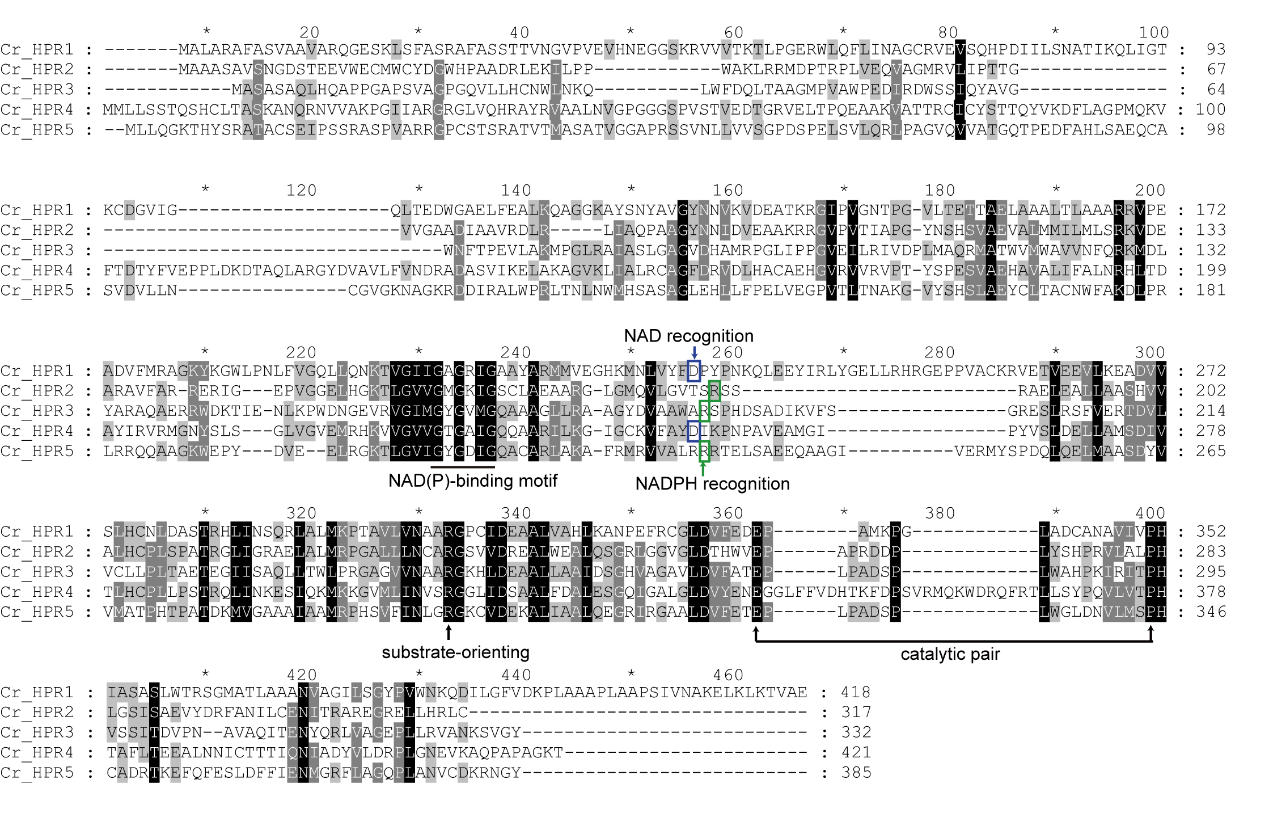


**Fig. S2.** **Sequence alignment of *Chlamydomonas* hydroxypyruvate reductase.**

Black highlighting indicates identical residues; Light gray highlighting indicates conserved residues with a value of above 60%. Gray highlighting indicates conserved residues with a value of above 80 %. Gaps introduced to optimize sequence alignment are indicated by hyphen. The number to the right of each protein sequence indicates the number of amino acids in the protein. The conserved domain, including NAD-recognition, NAD-binding motif, substrate orienting and catalytic pair, are indicated with blue rectangle box. Amino acid sequences are: CrHPR1: hydroxypyruvate reductase 1 of *Chlamydomonas reinhardtii* (Accession No. Cre06.g295450.t1.2); CrHPR2: hydroxypyruvate reductase 2 of *Chlamydomonas reinhardtii* (Accession No. Cre01.g019100.t1.2); CrHPR3: hydroxypyruvate reductase 3 of *Chlamydomonas reinhardtii* (Accession No. Cre02.g087300.t1.3); CrHPR4: hydroxypyruvate reductase 4 of *Chlamydomonas reinhardtii* (Accession No. Cre07.g324550.t1.3); CrHPR5: hydroxypyruvate reductase 5 of *Chlamydomonas reinhardtii* (Accession No. Cre16.g689700.t1.3). The sequences are aligned using ClustalW software.


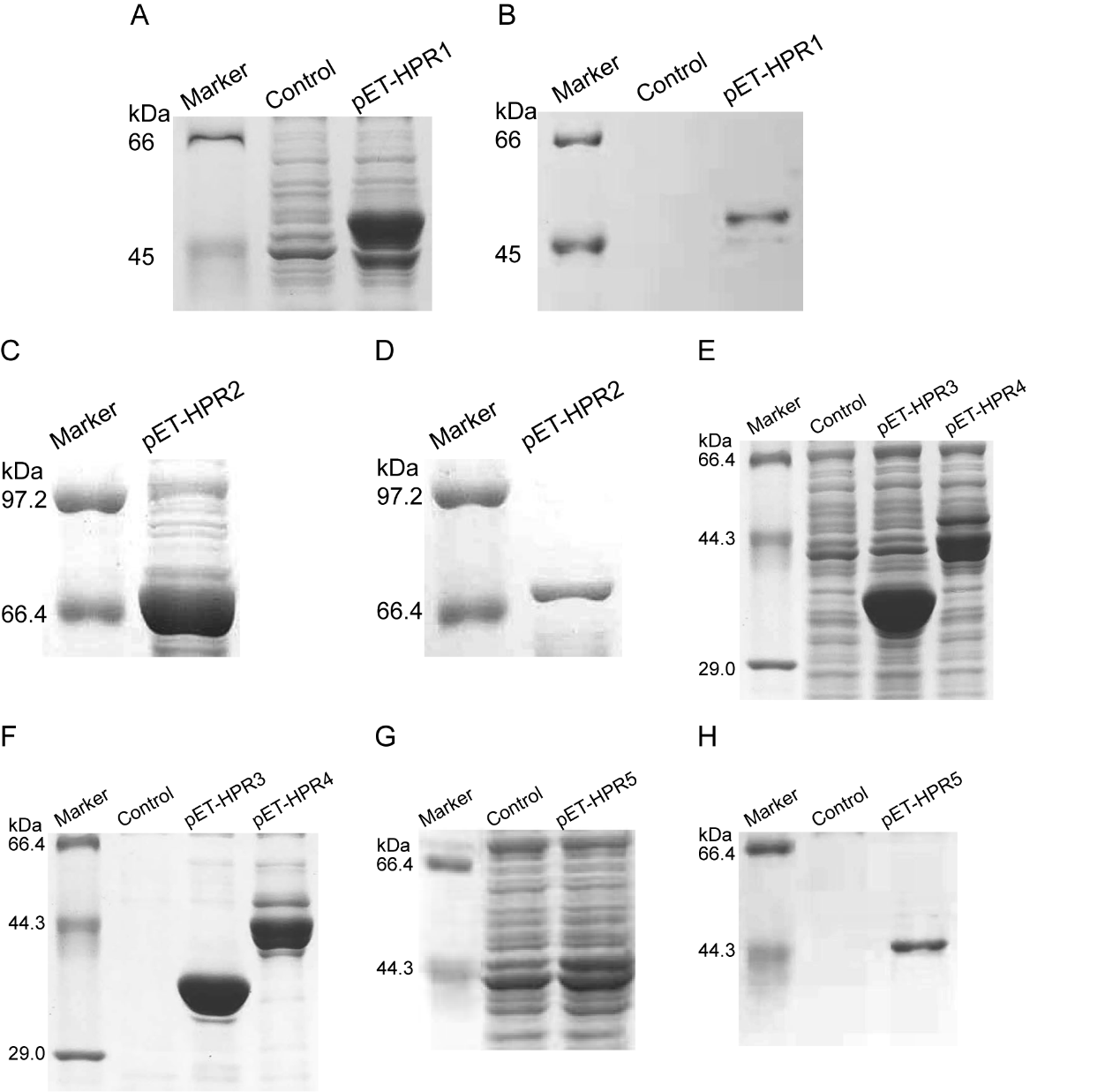


**Fig. S3.** **Expression and purification of recombinant CrHPRs assayed by SDS-PAGE gels**.

(A)(C)(E)(G), Gel imaging of His-tagged recombinant CrHPRs before affinity purification. Proteins were separated on SDS-PAGE (12%) and stained by Coomassie Brilliant Blue R.

(B)(D)(F)(H), Gel imaging of His-tagged recombinant CrHPRs after affinity purification. The recombinant CrHPRs were eluted with 100 mM imidazole and separated on SDS-PAGE (12%) with Coomassie Brilliant Blue R stained. Control strain were transformed by empty pET-30a vector.


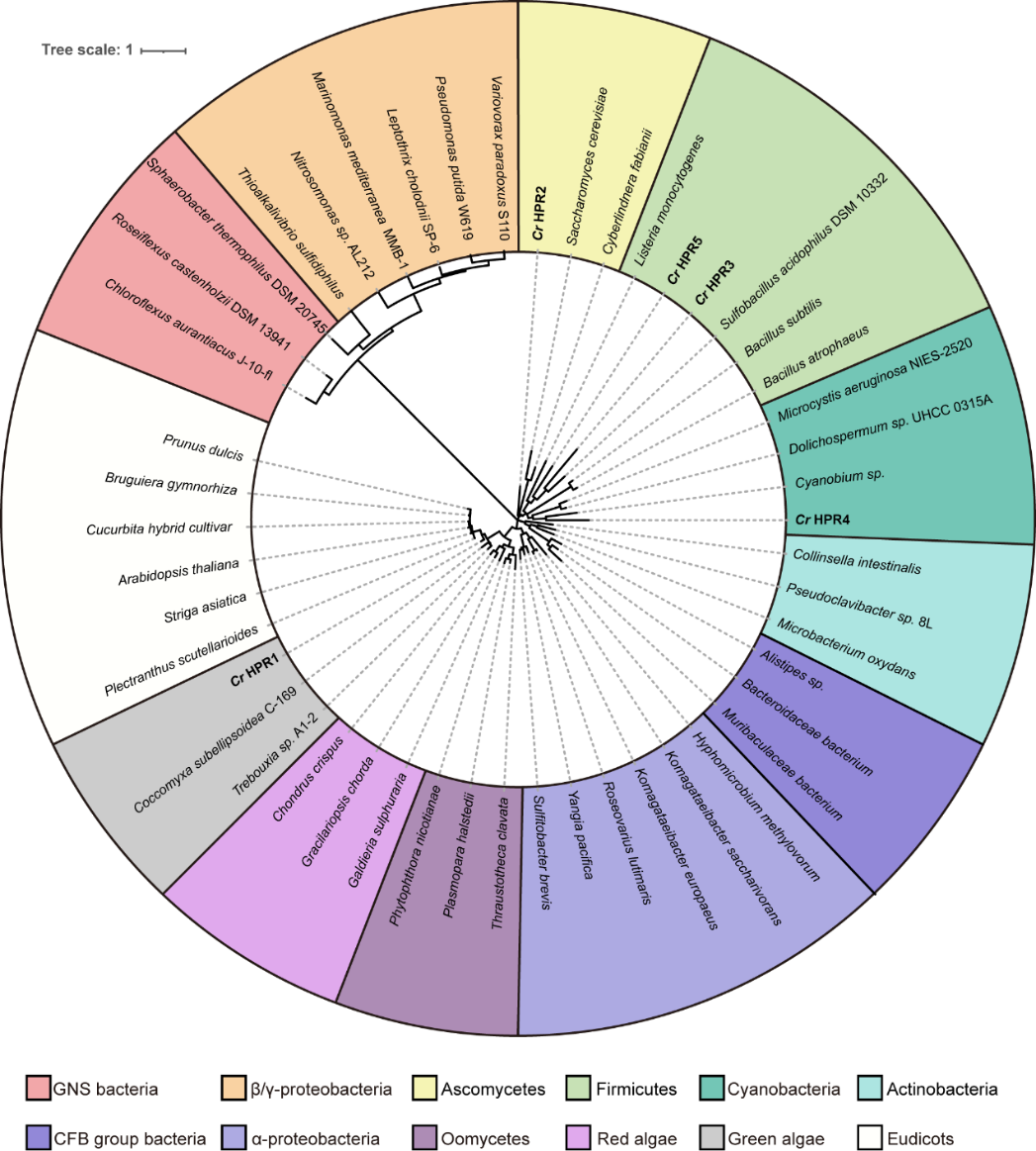


**Fig. S4.** **Phylogenetic tree of HPR proteins inferred by** **bacterial and eukaryotic sources.**

The tree was constructed using the maximum likelihood algorithm with 36 amino acid sequences (Kumar et al., 2015). The final dataset has a total of 140 locations. An evolutionary analysis was performed in MEGA7. Amino acid sequences are: *Alistipes sp.* (GFI53547.1); *Arabidopsis thaliana* (NP_001185349.1); *Bacillus atrophaeus* (KYD02573.1); *Bacillus subtilis* (RUS09252.1); *Bacteroidaceae bacterium* (GFI00459.1); *Bruguiera gymnorhiza* (BAB44155.1); *Chloroflexus aurantiacus* J-10-fl (ABY33859.1); *Chondrus crispus* (XP_005710458.1); *Coccomyxa subellipsoidea* C-169 (XP_005648728.1); *Collinsella intestinalis* (VWM19403.1); CrHPR1 (Cre06.g295450.t1.2); CrHPR2 (Cre01.g019100.t1.2); CrHPR3 (Cre02.g087300.t1.3); CrHPR4 (Cre07.g324550.t1.3); CrHPR5 (Cre16.g689700.t1.3); *Cucurbita hybrid cultivar* (BAA08411.1); *Cyanobium sp.* (GDX75577.1); *Cyberlindnera fabianii* (ONH69903.1); *Dolichospermum sp.* UHCC 0315A (QEI42781.1); *Galdieria sulphuraria* (EME27744.1); *Gracilariopsis chorda* (PXF44119.1); *Hyphomicrobium methylovorum* (BAA06662.1); *Komagataeibacter europaeus* (ARW16676.1); *Komagataeibacter saccharivorans* (PMP98341.1); *Leptothrix cholodnii* SP-6 (ACB35436.1); *Listeria monocytogenes* (CAB3444959.1); *Marinomonas mediterranea* MMB-1 (ADZ92092.1); *Microbacterium oxydans* (AZS48620.1); *Microcystis aeruginosa* NIES-2520 (GCA73651.1); *Muribaculaceae bacterium* (GFI14504.1); *Nitrosomonas sp.* AL212 (ADZ25632.1); *Phytophthora nicotianae* (KUF78562.1); *Plasmopara halstedii* (XP_024573522.1); *Plectranthus scutellarioides* (ABL10359.1); *Prunus dulcis* (BBH09815.1); *Pseudoclavibacter sp.* 8L (VXB05721.1); *Pseudomonas putida* W619 (ACA74107.1); *Roseiflexus castenholzii* DSM 13941 (ABU59323.1); *Roseovarius lutimaris* (SFN91301.1); *Saccharomyces cerevisiae* (ONH81010.1); *Sphaerobacter thermophilus* DSM 20745 (ACZ37940.1); *Striga asiatica* (GER35896.1); *Sulfitobacter brevis* (SFD51819.1); *Sulfobacillus acidophilus* DSM 10332 (AEW04239.1); *Thioalkalivibrio sulfidiphilus* (ACL72019.1); *Thraustotheca clavate* (OQR99548.1); *Trebouxia sp.* A1-2 (KAA6422491.1); *Variovorax paradoxus* S110 (ACS20535.1); *Yangia pacifica* (SFS73526.1).


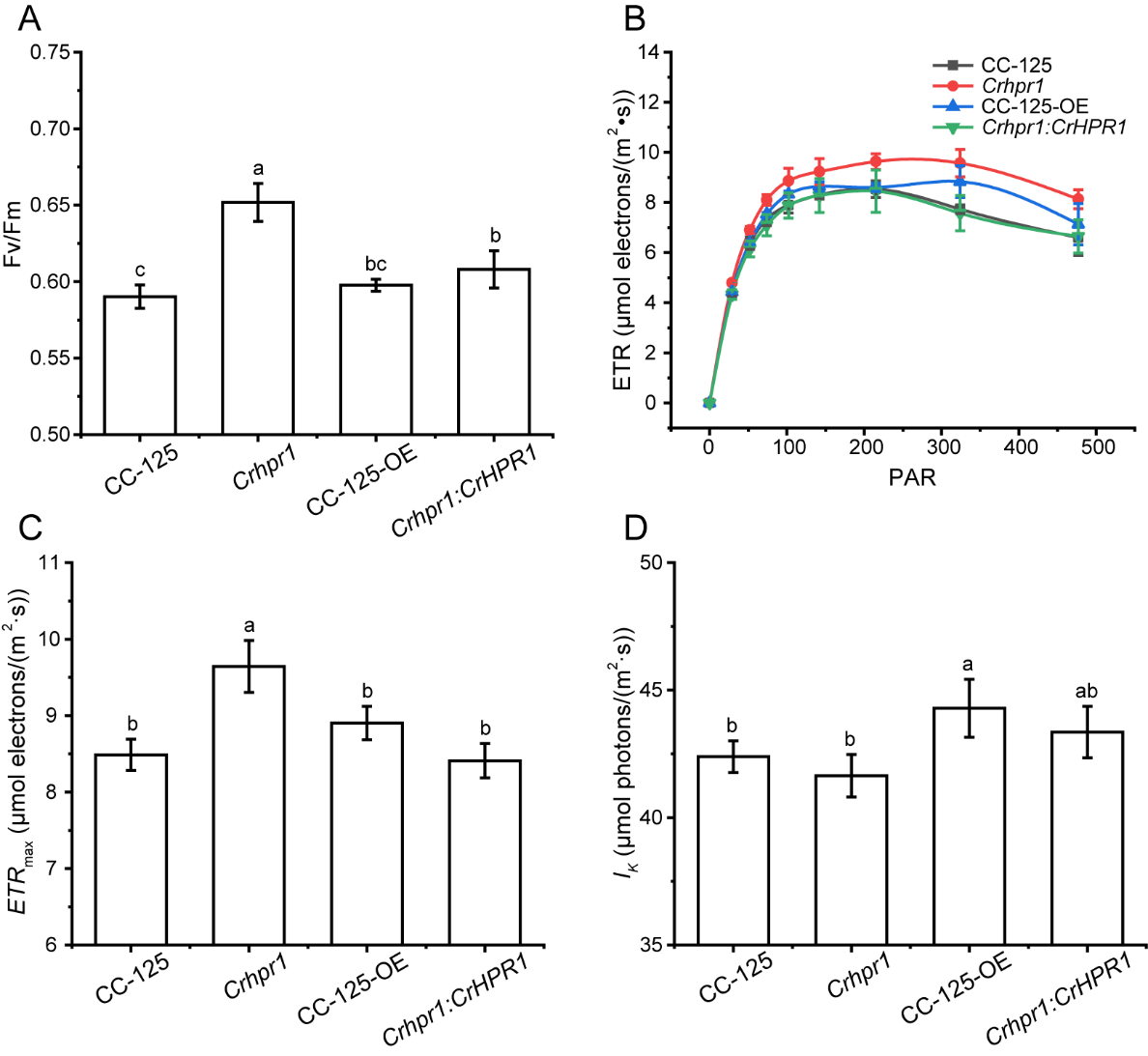


**Fig. S5.** **Measurement of photosynthetic activity of the *Crhpr1* and the rescued strains by chlorophyll fluorescence**. (A) Maximum photochemical quantum yield of PSII. (B) Electron transport rate (ETR). (C) Maximum electron transfer efficiency. (D) Minimum saturating irradiance. Mean values ±SD from three measurements. Means denoted by the same letter did not significantly differ at P<0.05.


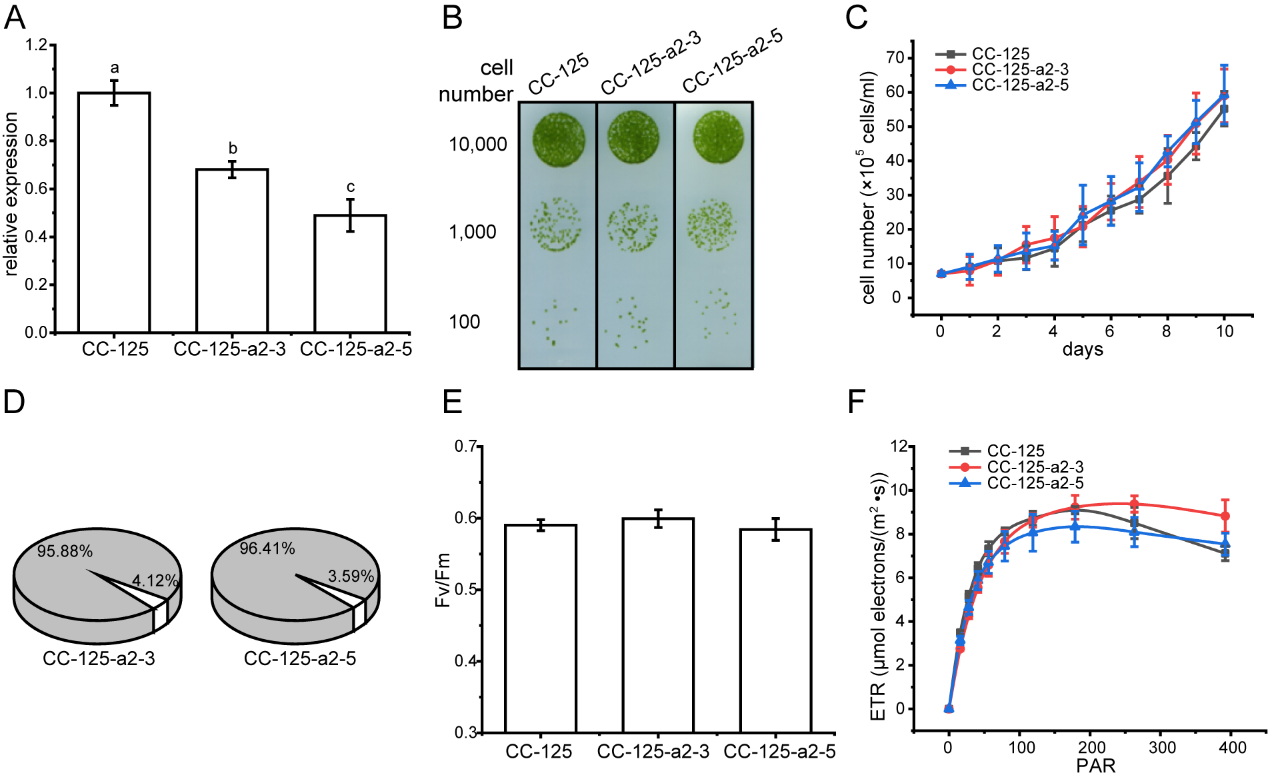


**Fig. S6. Phenotypic analysis of the *CrHPR2* knockdown strains at CC-125 background.** (A) Measurement of *CrHPR2* transcripts in CC-125-a2 strains. (B) Spot tests showing growth of CC-125 and CC-125-a2 strains. (C) Growth curves of CC-125 and CC-125-a2 strains. (D) The ratio of oxidation and carboxylation reaction of Rubisco in CC-125-a2 strains. Carboxylation reaction: white sector, Oxidation reaction: gray sector. (E) Maximum photochemical quantum yield of PSII. (F) Electron transport rate (ETR). Mean values ±SD from three measurements. Means denoted by the same letter did not significantly differ at P<0.05.


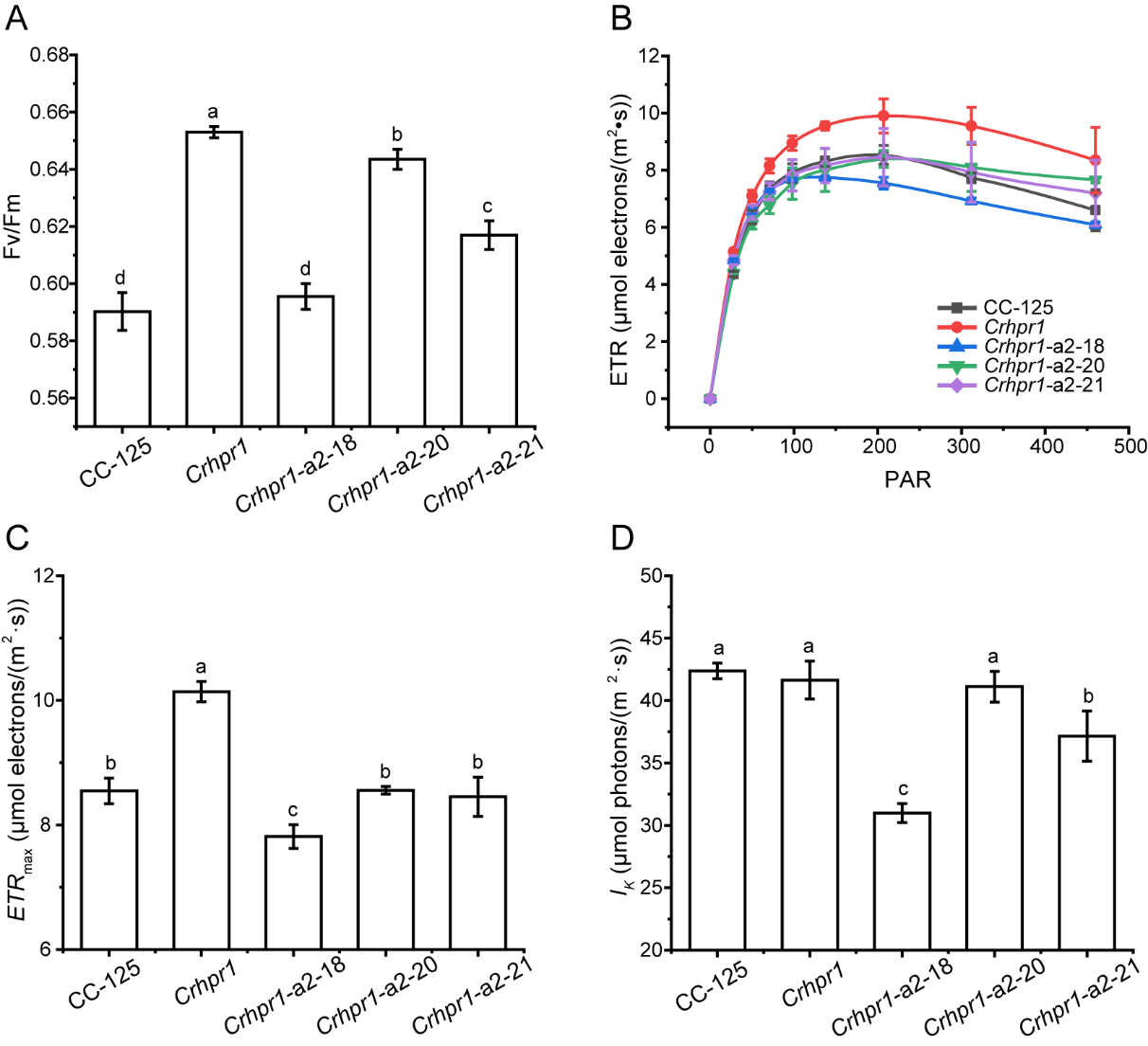


**Fig. S7.** **Measurement of photosynthetic activity of *Crhpr1-a2* strains by chlorophyll fluorescence.** (A) Maximum photochemical quantum yield of PSII. (B) Electron transport rate (ETR). (C) Maximum electron transfer efficiency. (D) Minimum saturating irradiance. Mean values ±SD from three measurements. Means denoted by the same letter did not significantly differ at P<0.05.


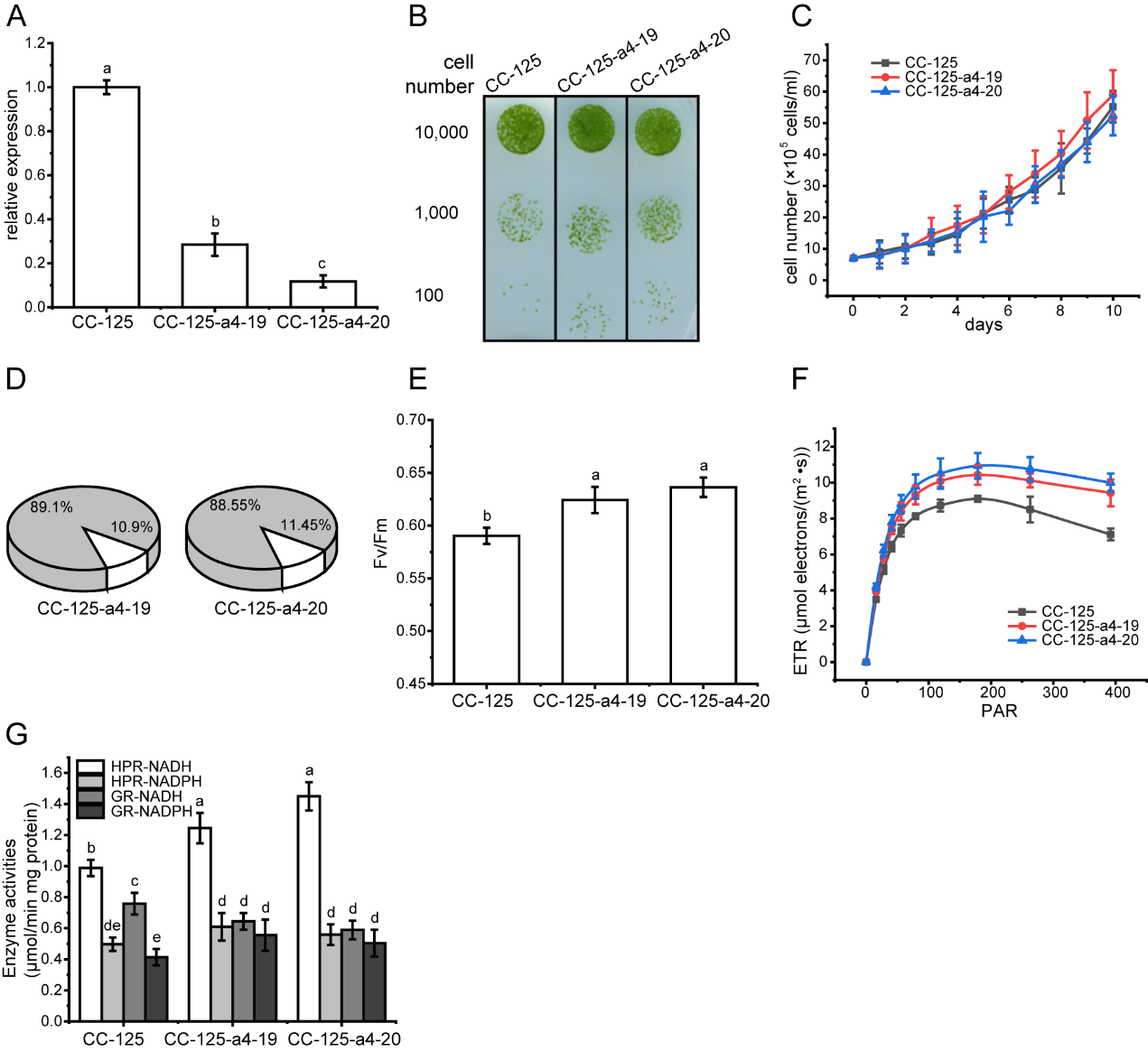


**Fig. S8. Phenotypic analysis of the *CrHPR4* knockdown strains at CC-125 background.** (A) Measurement of *CrHPR4* transcripts in CC-125-a4 strains. (B) Spot tests showing growth of CC-125 and CC-125-a4 strains. (C) Growth curves of CC-125 and CC-125-a4 strains. (D) The ratio of oxidation and carboxylation reaction of Rubisco in CC-125-a4 strains. Carboxylation reaction: white sector, Oxidation reaction: gray sector. (E) Maximum photochemical quantum yield of PSII. (F) Electron transport rate (ETR). (G) Enzyme activity assay of CC-125 and CC-125-a4 strains. Mean values ±SD from three measurements. Means denoted by the same letter did not significantly differ at P<0.05.


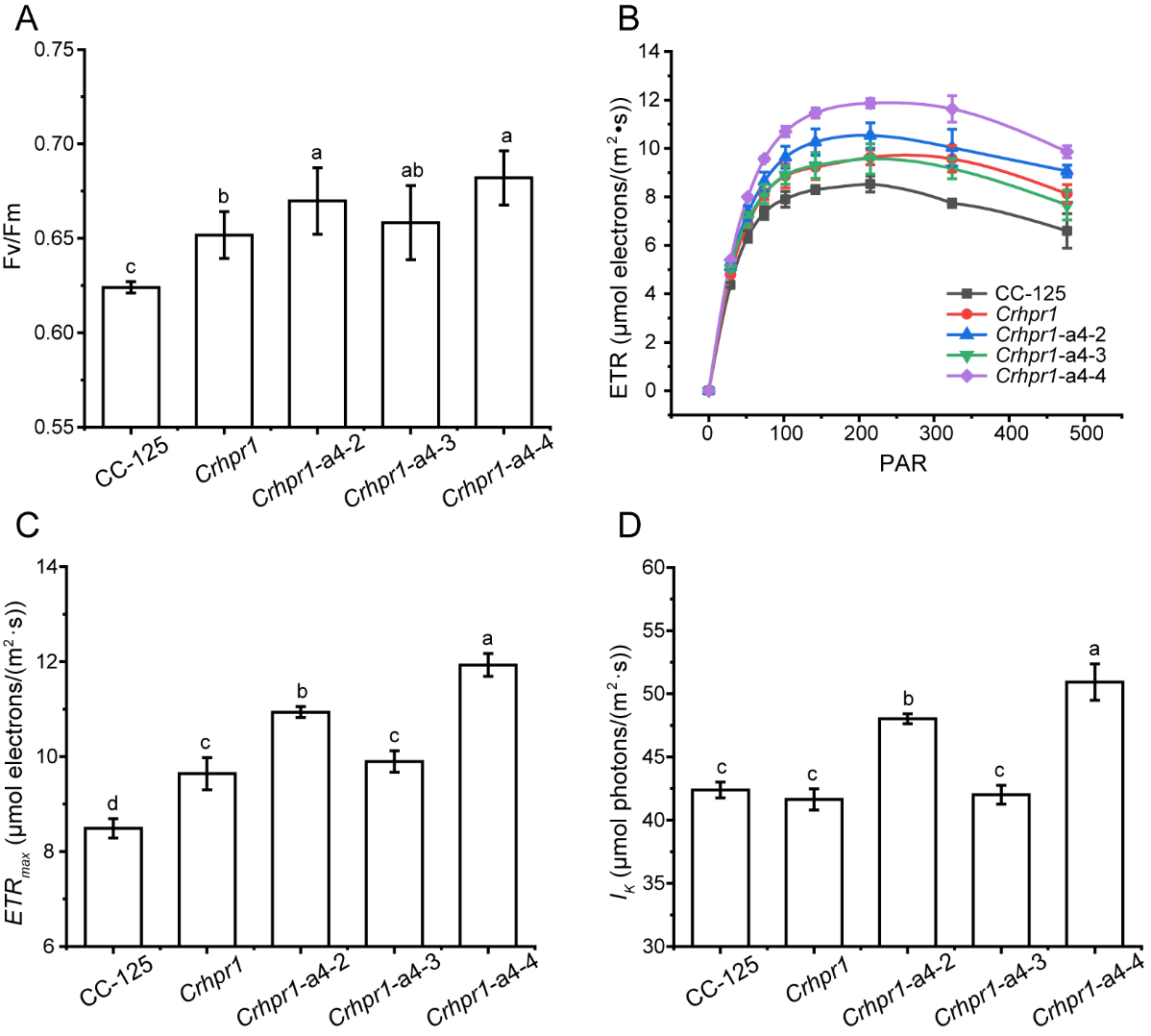


**Fig. S9.** **Measurement of photosynthetic activity of *Crhpr1-a4* strains by chlorophyll fluorescence.** (A) Maximum photochemical quantum yield of PSII. (B) Electron transport rate (ETR). (C) Maximum electron transfer efficiency. (D) Minimum saturating irradiance. Mean values ±SD from three measurements. Means denoted by the same letter did not significantly differ at P<0.05.
